# Supplementary figures and images for: Factors enforcing the species boundary between the human pathogens Cryptococcus neoformans and Cryptococcus deneoformans
Source: PLoS Genet. 2021 Jan 19;17(1):e1008871. doi: 10.1371/journal.pgen.1008871 (PMC7846113; doi:10.1371/journal.pgen.1008871)

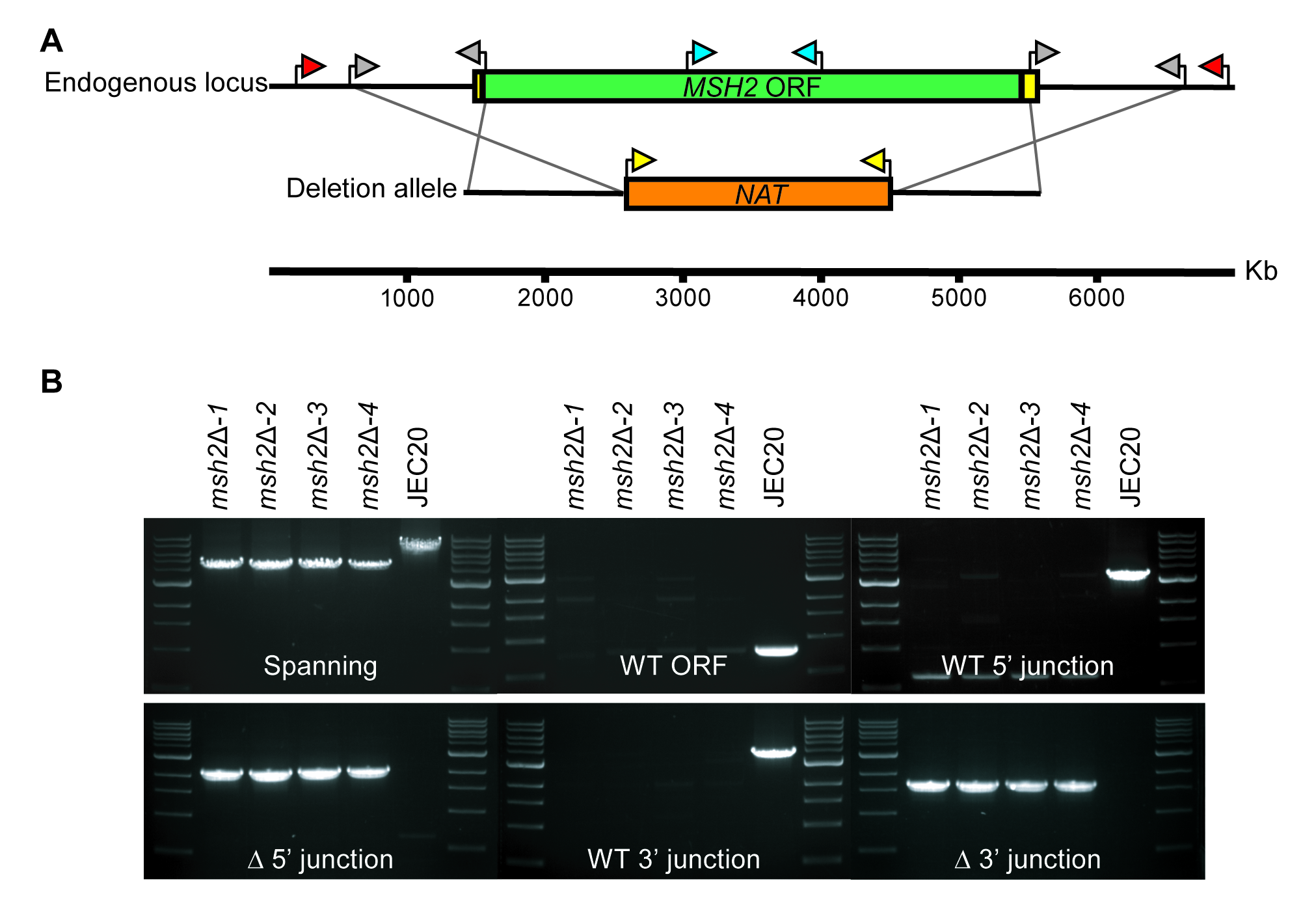

Supplement: S1 Fig — (A) Genetic deletion mutants lacking MSH2 were engineered in JEC20a by replacing the MSH2 open reading frame (ORF) with the dominant drug resistance marker NAT which confers resistance to nourseothricin via biolistic transformation. 5’ and 3’ UTRs are depicted as yellow boxes. Arrows depict locations of primers used to generate and verify MSH2 deletion. The sets of primers denoted by gray arrows located upstream and downstream of the MSH2 ORF were used to amplify flanking sequences homologous to the JEC20a MSH2 endogenous locus to mediate homologous recombination (JOHE45551, 45552 and JOHE4555, 45556, respectively). Yellow arrows depict primers (JOHE45553 and JOHE45554) that amplified the NAT cassette and share homology with JOHE45552 and JOHE45556, respectively. The red arrows (JOHE45559,45560) and blue arrows (JOHE45822,45823) indicate primers that confirmed integration of the deletion allele and loss of the MSH2 ORF, respectively. Gray lines indicate syntenic regions shared between the deletion allele and endogenous locus. (B) Gel electrophoresis of PCR products was used to confirm integration of a single copy of the NAT gene at the correct locus and that the wild-type MSH2 gene was absent in the JEC20a mutant strains. (Spanning: JOHE45559,45560; WT in-gene: JOHE45822,45823; WT 5’ junction: JOHE45559,45823; Δ5’ junction: JOHE45559;45554; WT 3’ junction: JOHE45822,45560; Δ3’ junction: JOHE45553,45560). (TIF) [file pgen.1008871.s011.tif]

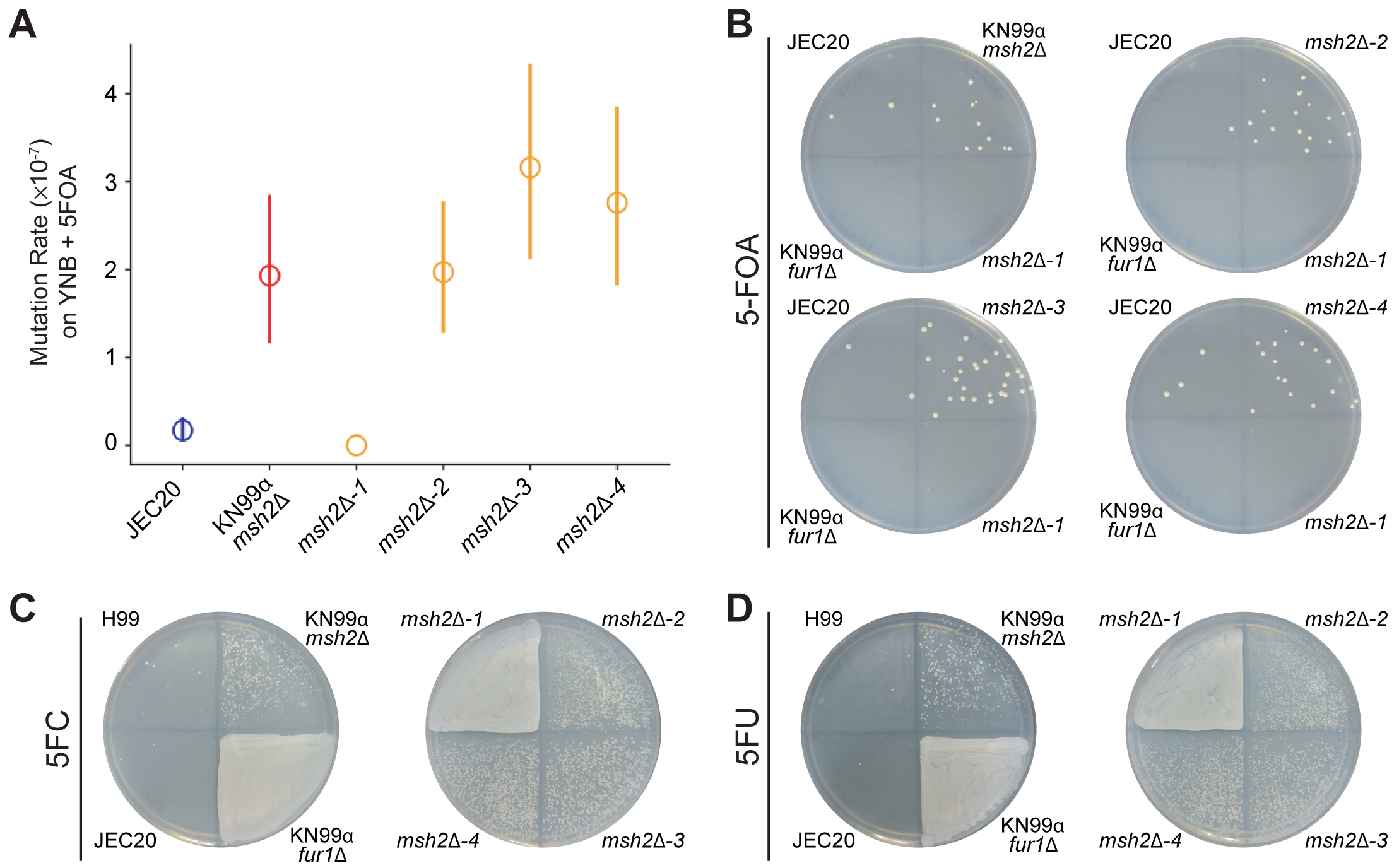

Supplement: S2 Fig — (A) Fluctuation analysis on YNB+5-FOA medium was performed in a similar manner to the analysis described in Fig 1A. (B-D) Representative images of plates used in papillation assays with independent JEC20a msh2Δ mutants and a KN99α fur1Δ mutant on (B) YNB+5-FOA medium, (C) YNB+5FC medium, and (D) YNB+5FU medium with the JEC20a parental strain and a KN99α msh2Δ mutant as controls. Strains were incubated on YNB+5-FOA medium for 6 days at 30°C before imaging. Strains were incubated on YNB+5FC and YNB+5FU media for 3 days at 30°C before imaging. (TIF) [file pgen.1008871.s012.tif]

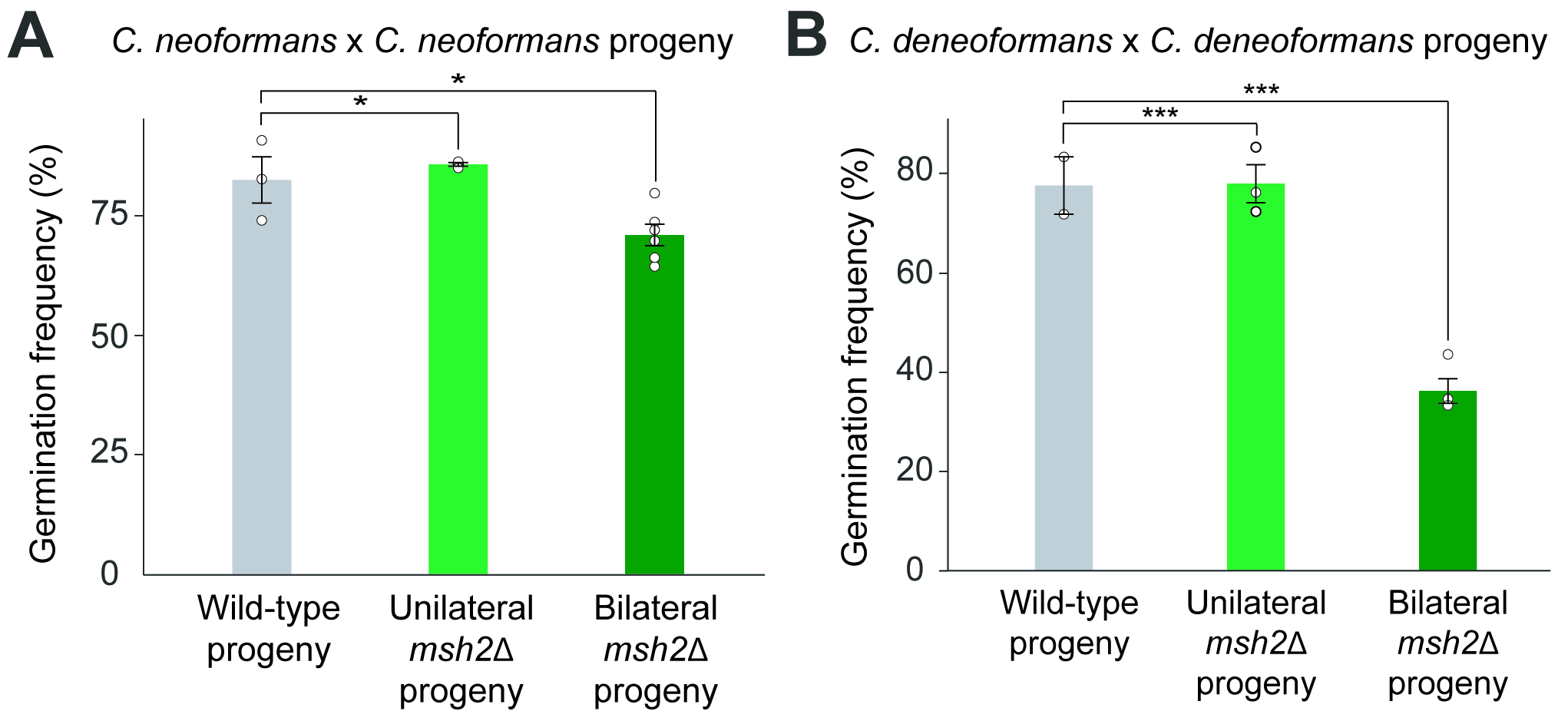

Supplement: S3 Fig — Average germination frequencies of progeny derived from (A) C. neoformans H99α x C. neoformans KN99a wild-type, unilateral msh2Δ (KN99α msh2Δ x KN99a), and bilateral msh2Δ crosses (KN99α msh2Δ x KN99a msh2Δ-1 and KN99α msh2Δ x KN99a msh2Δ-2), and (B) C. deneoformans JEC21α x C. deneoformans JEC20a wild-type, unilateral msh2Δ (JEC21α x JEC20a msh2Δ-1), and bilateral msh2Δ crosses (JEC21α msh2Δ-1 x JEC20a msh2Δ-1 and JEC21α msh2Δ-2 x JEC20a msh2Δ-1). Error bars represent standard error of the mean. Statistical significance was determined with one-way ANOVA and Tukey’s post hoc test. * indicates p<0.05, ** indicates p<0.01, and *** indicates p<0.001. (TIF) [file pgen.1008871.s013.tif]

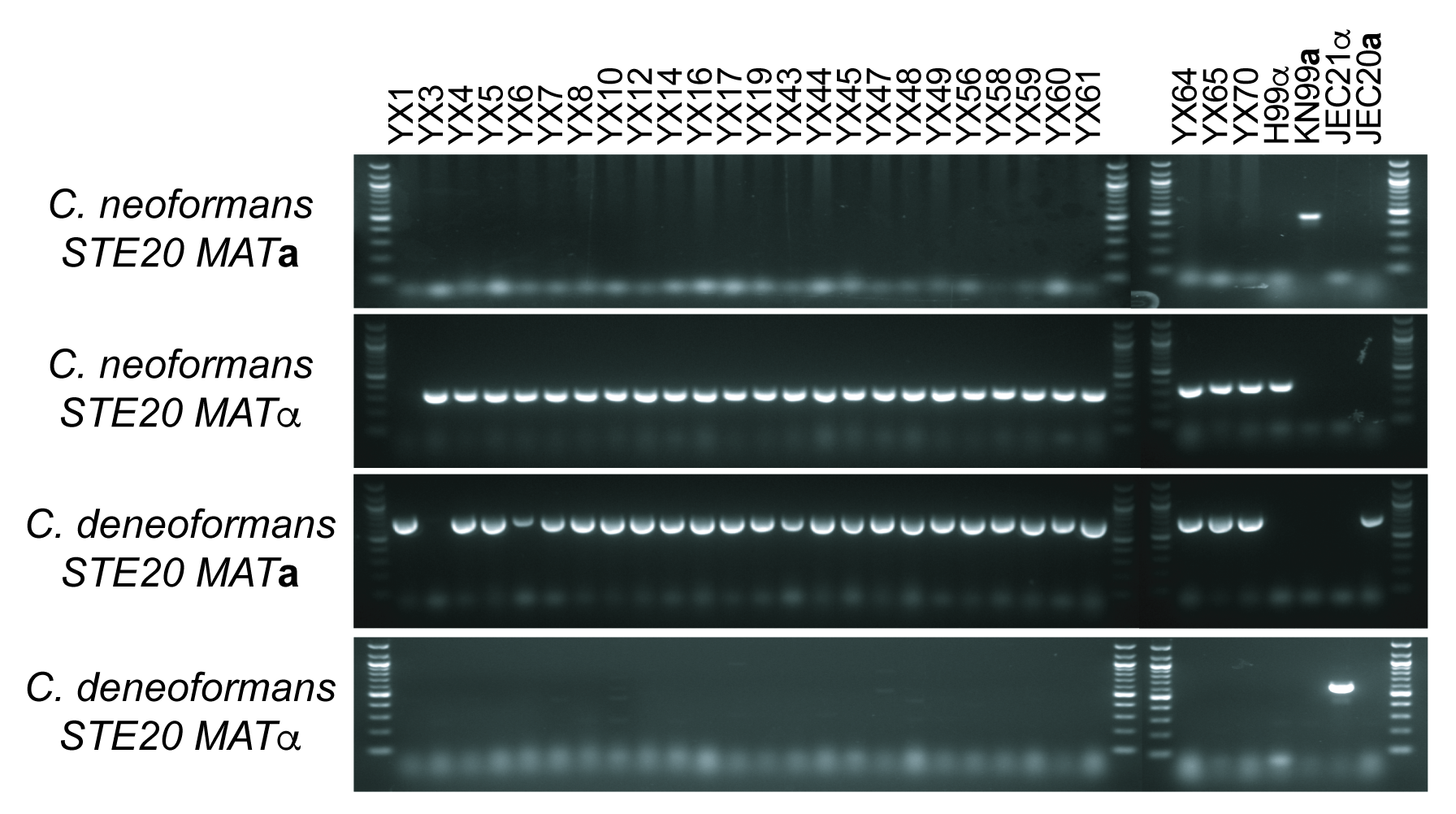

Supplement: S4 Fig — Sequence-specific primers for the mating-type (MAT) locus gene STE20, which can differentiate between C. neoformans MATa, C. neoformans MATα, C. deneoformans MATa, and C. deneoformans MATα, were used to characterize which MAT alleles each of the hybrid progeny inherited. (TIF) [file pgen.1008871.s014.tif]

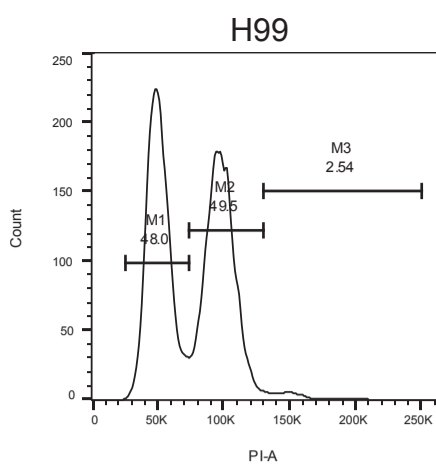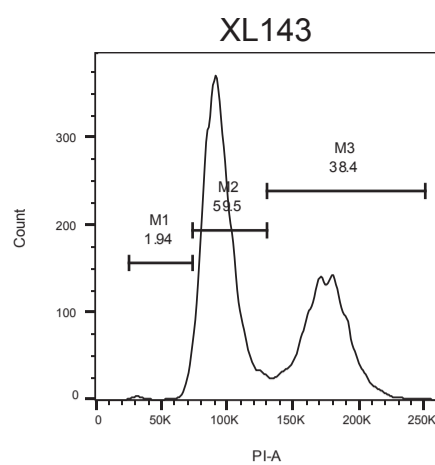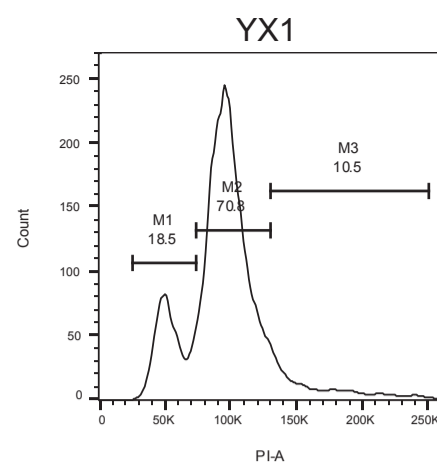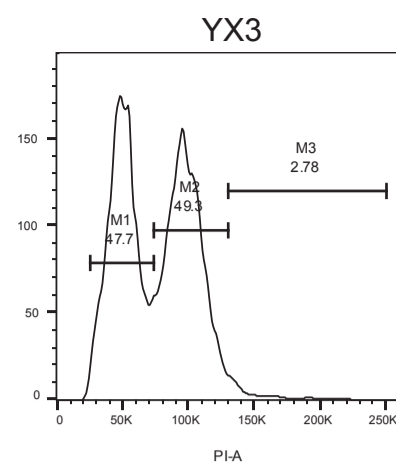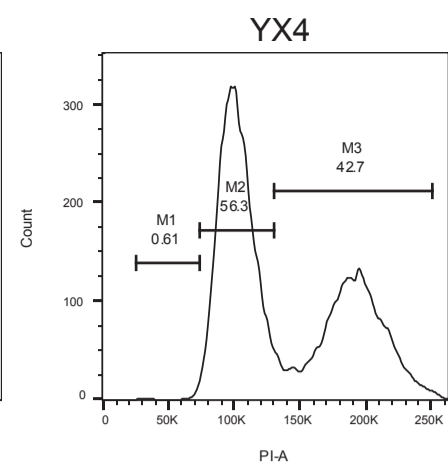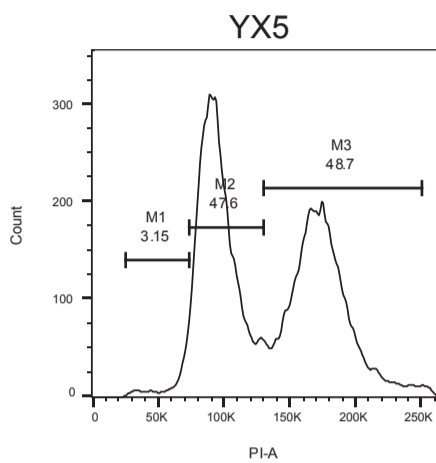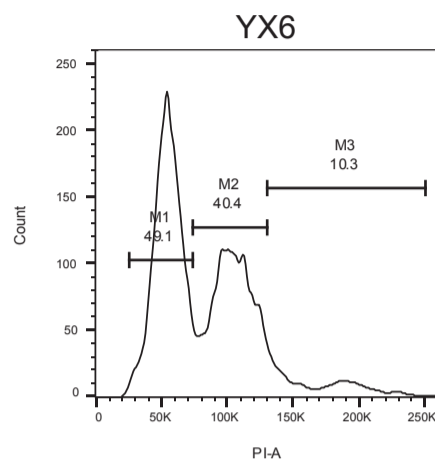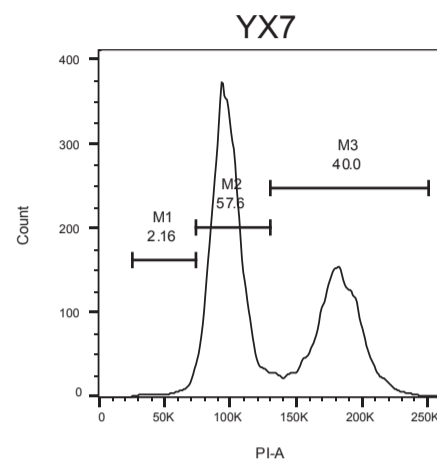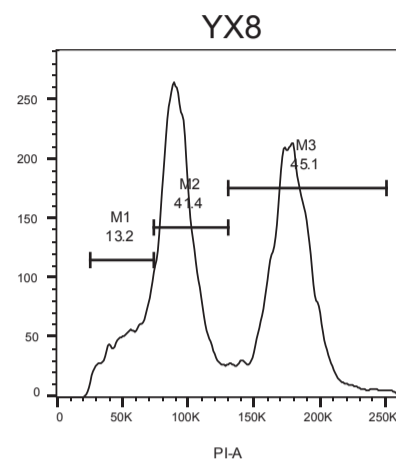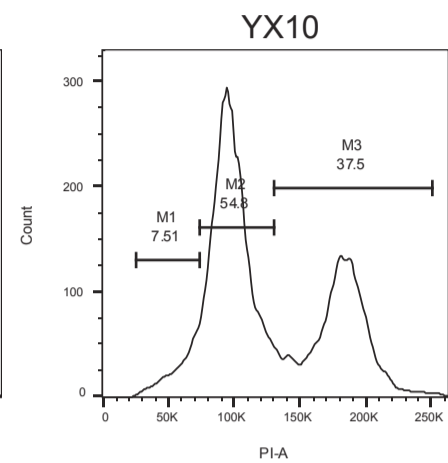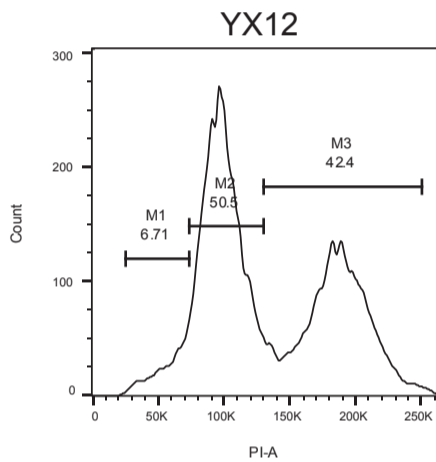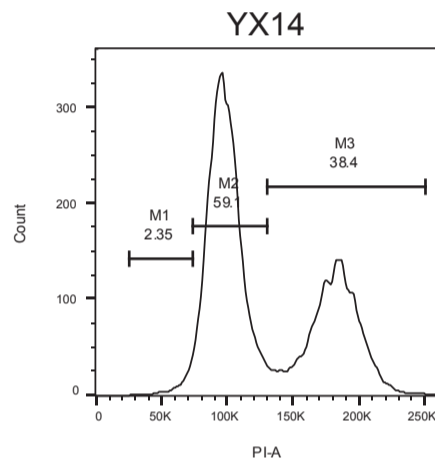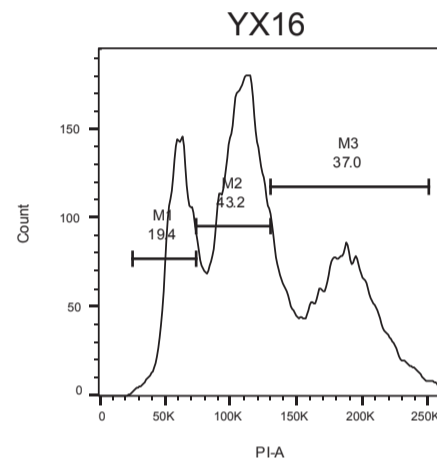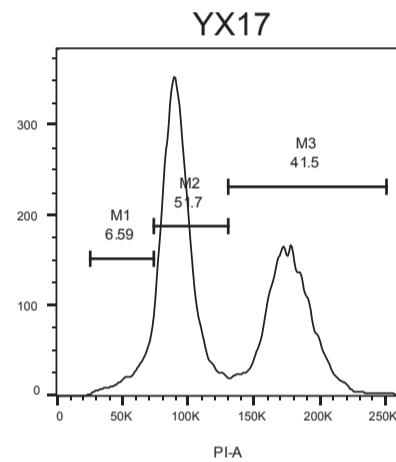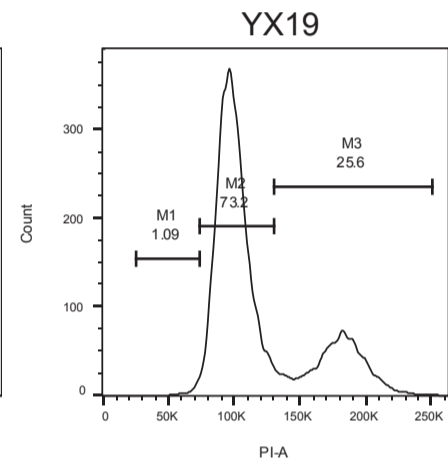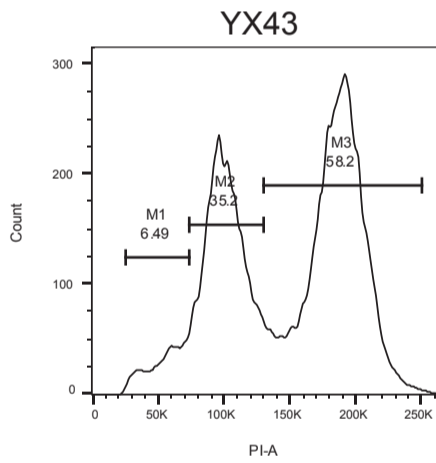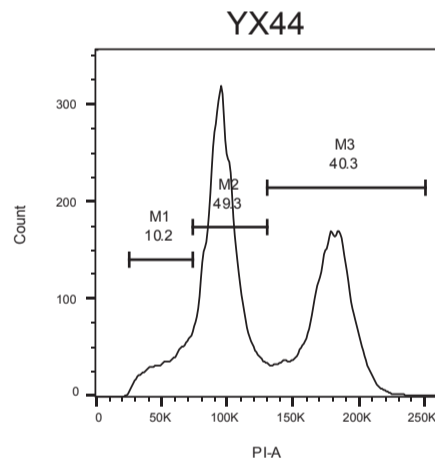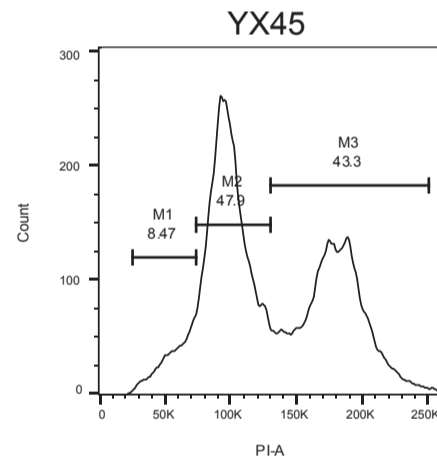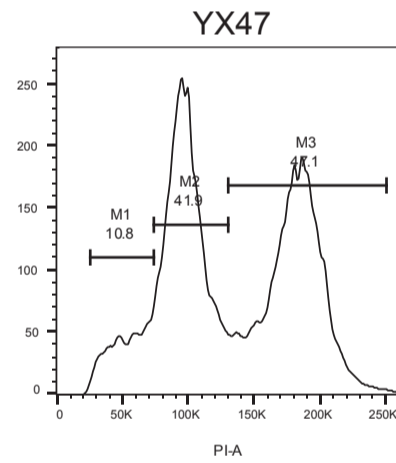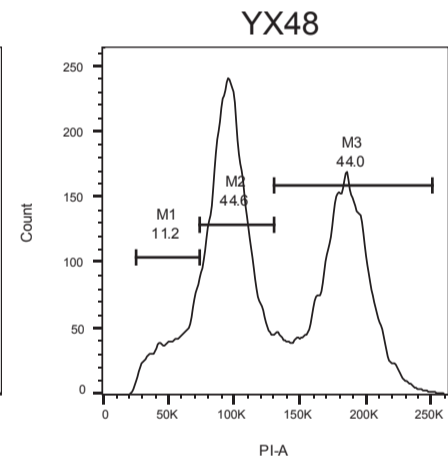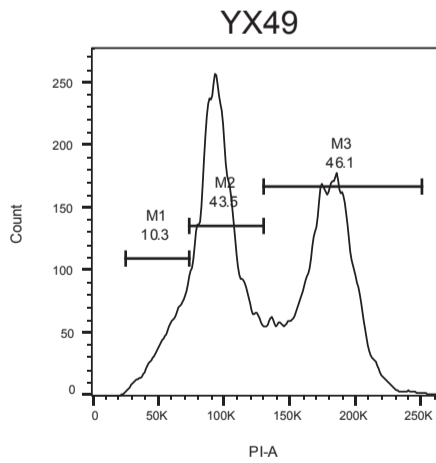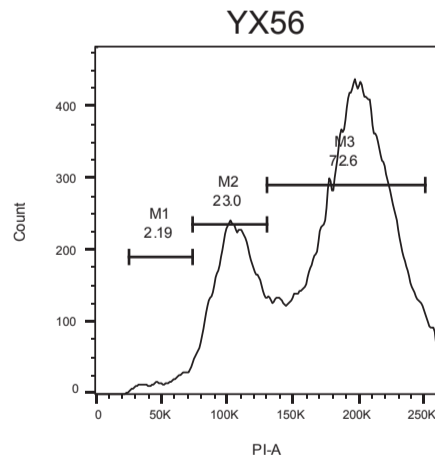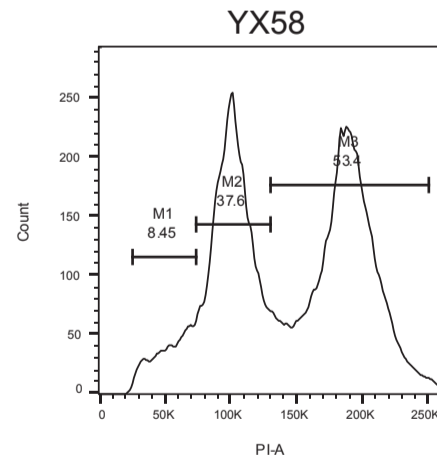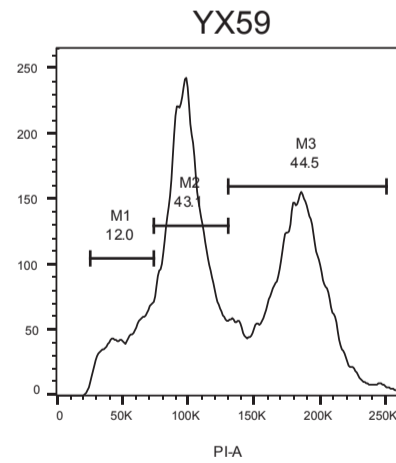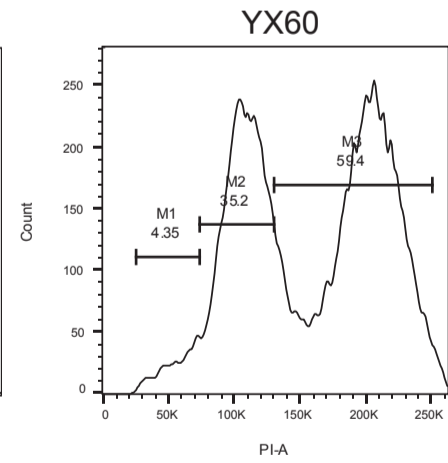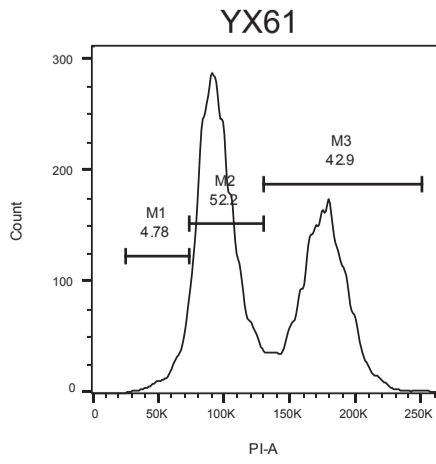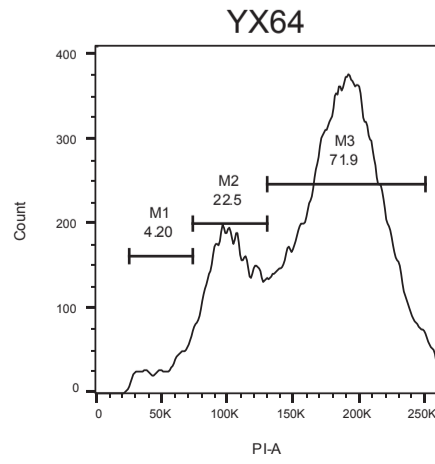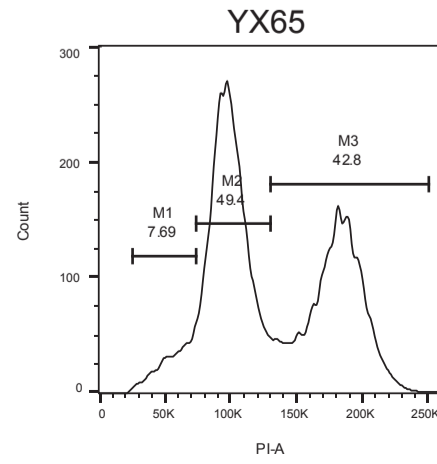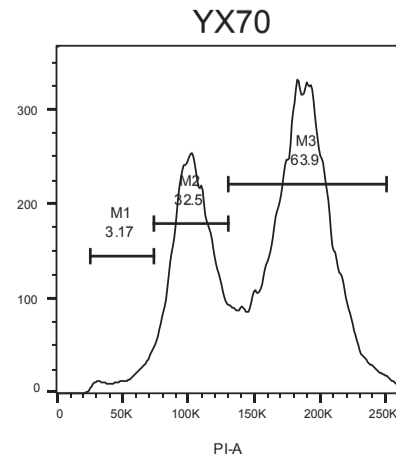

Supplement: S5 Fig — The C. neoformans strain H99α was used as a 1n haploid control and the C. deneoformans strain XL143 [65] was used as a diploid 2n control. (PDF) [file pgen.1008871.s015.pdf]

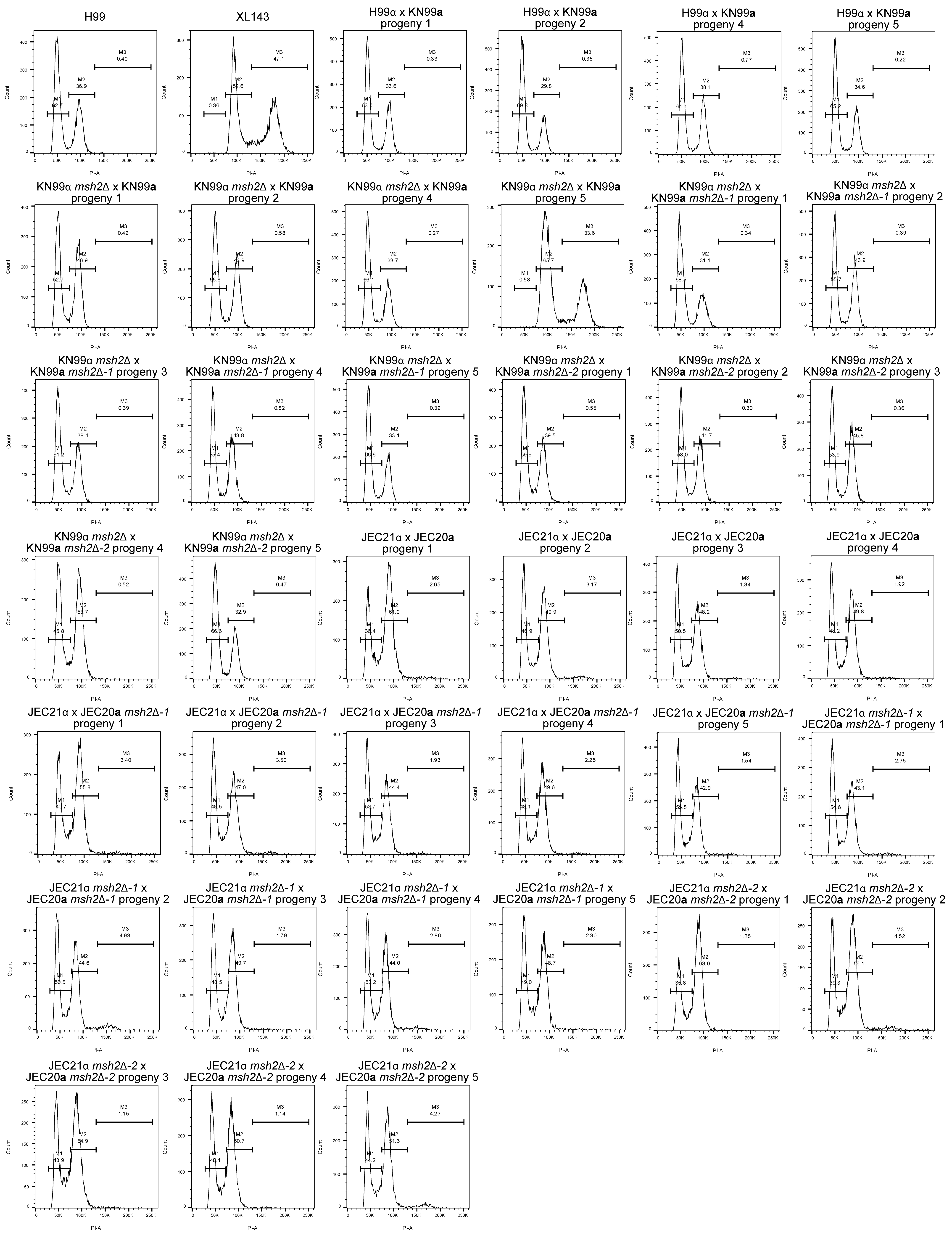

Supplement: S6 Fig — The C. neoformans strain H99α was used as a 1n haploid control and the C. deneoformans strain XL143 [65] was used as a diploid 2n control. (TIF) [file pgen.1008871.s016.tif]

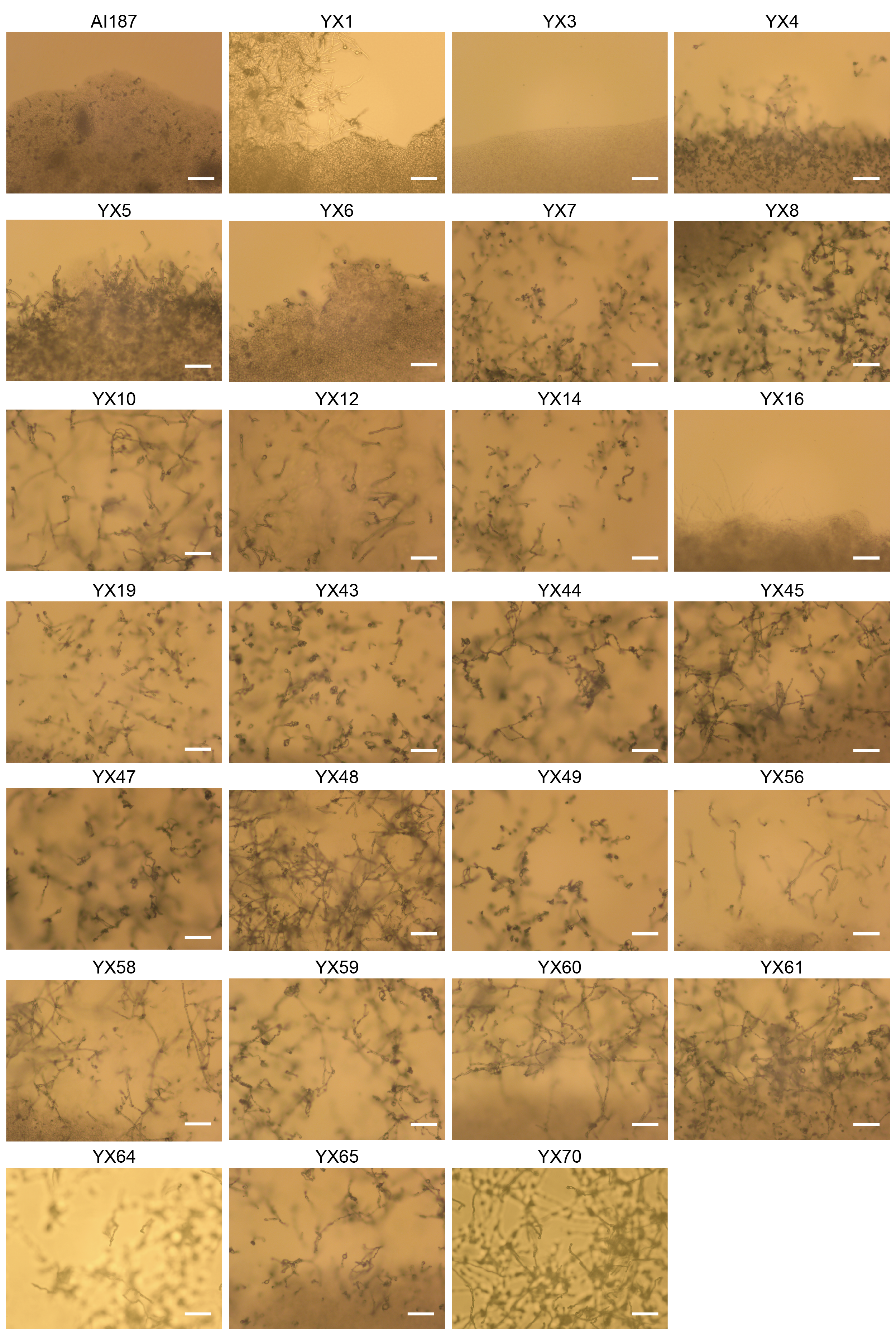

Supplement: S7 Fig — Filamentation of hybrid progeny selected for whole-genome sequencing on MS medium after incubation for 14 days. AI187 is a self-filamentous, stable diploid C. neoformans strain [103] and served as a positive control for production of hyphae. Scale bars represent 100 μm. (PNG) [file pgen.1008871.s017.png]

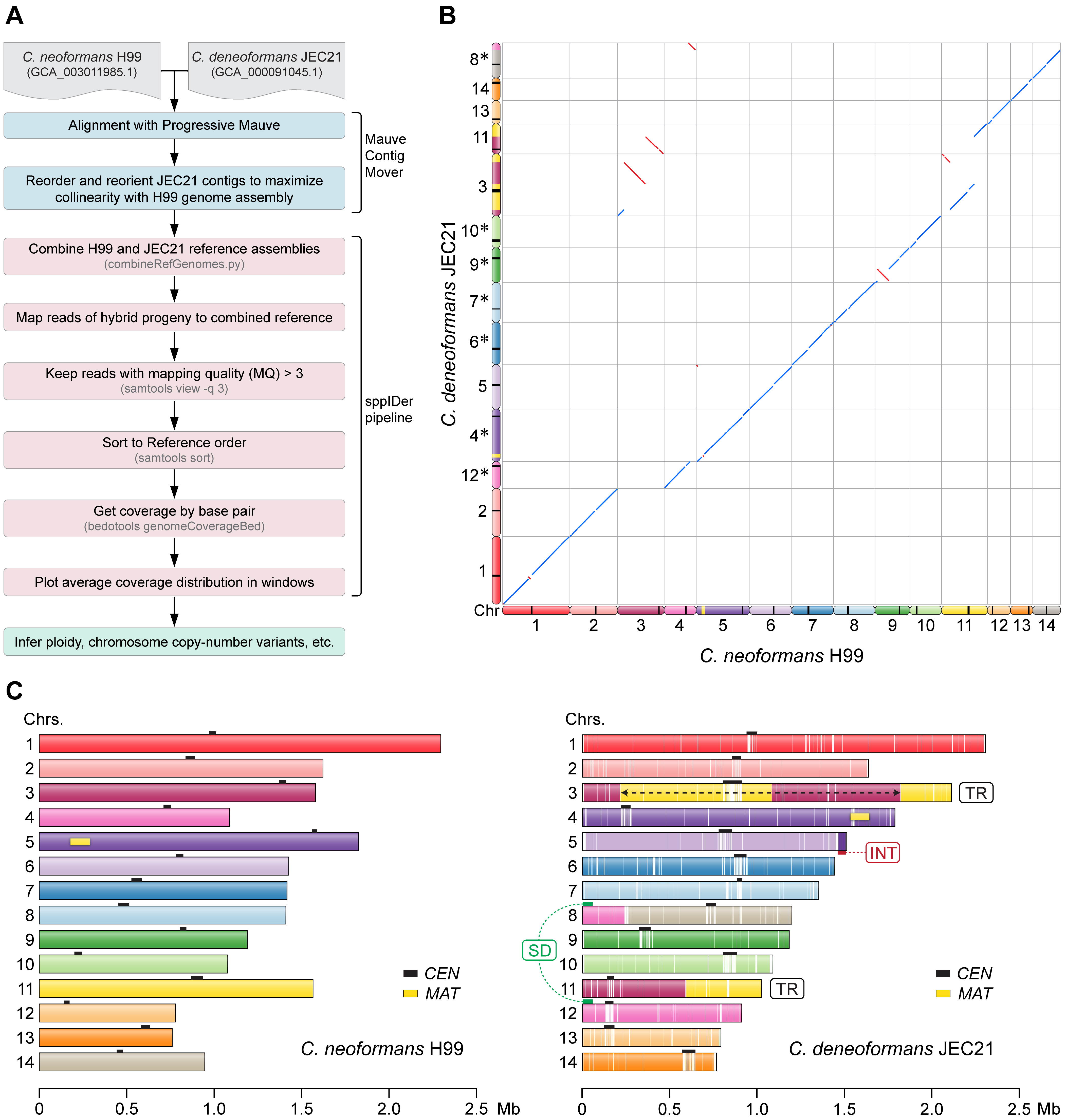

Supplement: S8 Fig — (A) Workflow to assess the genomic contribution of each parental species in the hybrid progeny. The sppIDer pipeline uses short-read sequencing data and a combined genome built from reference genomes of the two parental Cryptococcus species. (B) Dot-plot comparing the H99α assembly with the JEC21α reordered and reoriented assembly. Blue and red lines represent sequences with high similarities in the same and reverse orientations, respectively. (C) Linear plots showing overall synteny between the H99α and JEC21α genomes. The chromosomal positions of centromeres and the MAT locus are indicated by black and yellow bars, respectively. Chromosomes of JEC21α are color coded based on their synteny with the H99α chromosomes. Three major gross chromosomal changes previously documented distinguishing the two strains correspond to color changes within the same chromosome: TR indicates a reciprocal chromosomal translocation; INT indicates an introgression of a 14-gene region from C. neoformans to C. deneoformans that was mediated by transposable elements common to both lineages [27]; and SD indicates a segmental duplication following a nonreciprocal translocation involving the subtelomeric regions of JEC21 chromosomes 8 and 12 that presumably occurred during the construction of the congenic strain pair JEC21α/JEC20a [104]. Chromosomal inversions are not indicated except for a large inversion on Chr3 of JEC21α (dashed arrow); see [42] for more detailed descriptions of inversions. (TIF) [file pgen.1008871.s018.tif]

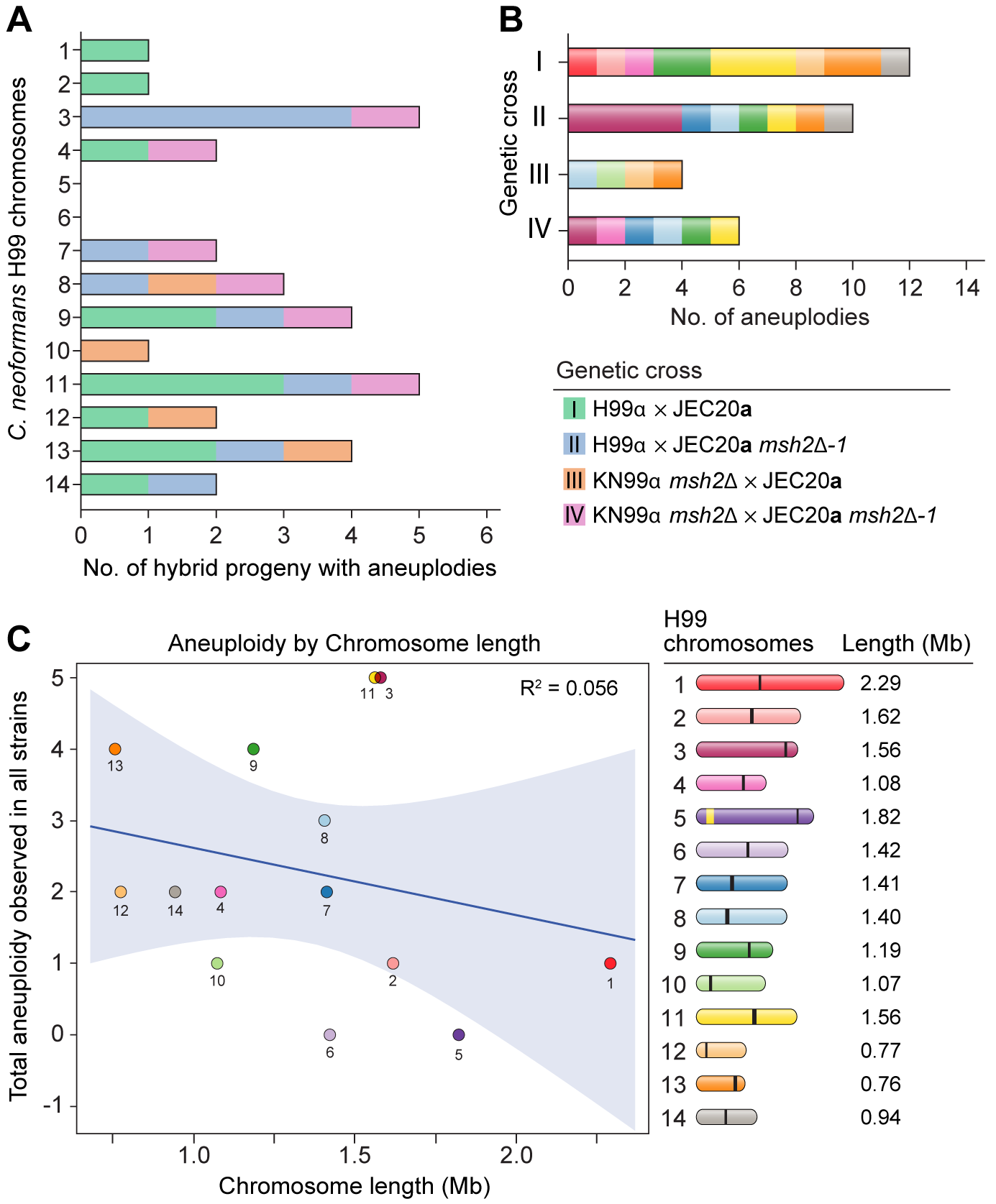

Supplement: S10 Fig — Graphs depicting the number of hybrid progeny with aneuploidies for each chromosome (A) and genetic cross (B). Graph showing no correlation between whole-chromosome aneuploidy events observed (y-axis) and chromosomal size (x-axis). Blue line represents linear fit and blue shaded area represents the 95% confidence interval for the fitted line. (TIF) [file pgen.1008871.s020.tif]

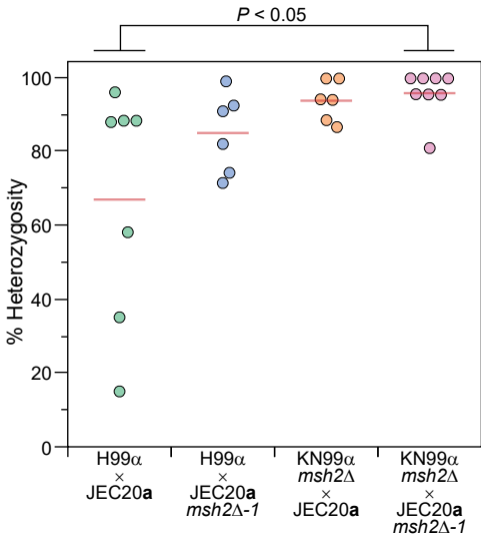

Supplement: S11 Fig — Plot showing the percentage of heterozygosity for each individual progeny (represented by different dots) grouped by type of cross (see S5 Table for details). The horizontal red line depicts the mean heterozygosity values. Genomes of progeny derived from bilateral msh2Δ × msh2Δ mutant crosses were significantly more heterozygous only when compared to hybrid progeny derived from wild-type H99α x JEC20a crosses (Statistical analysis: Kruskal-Wallis test, followed by Dunn’s test, p<0.05). (PDF) [file pgen.1008871.s021.pdf]

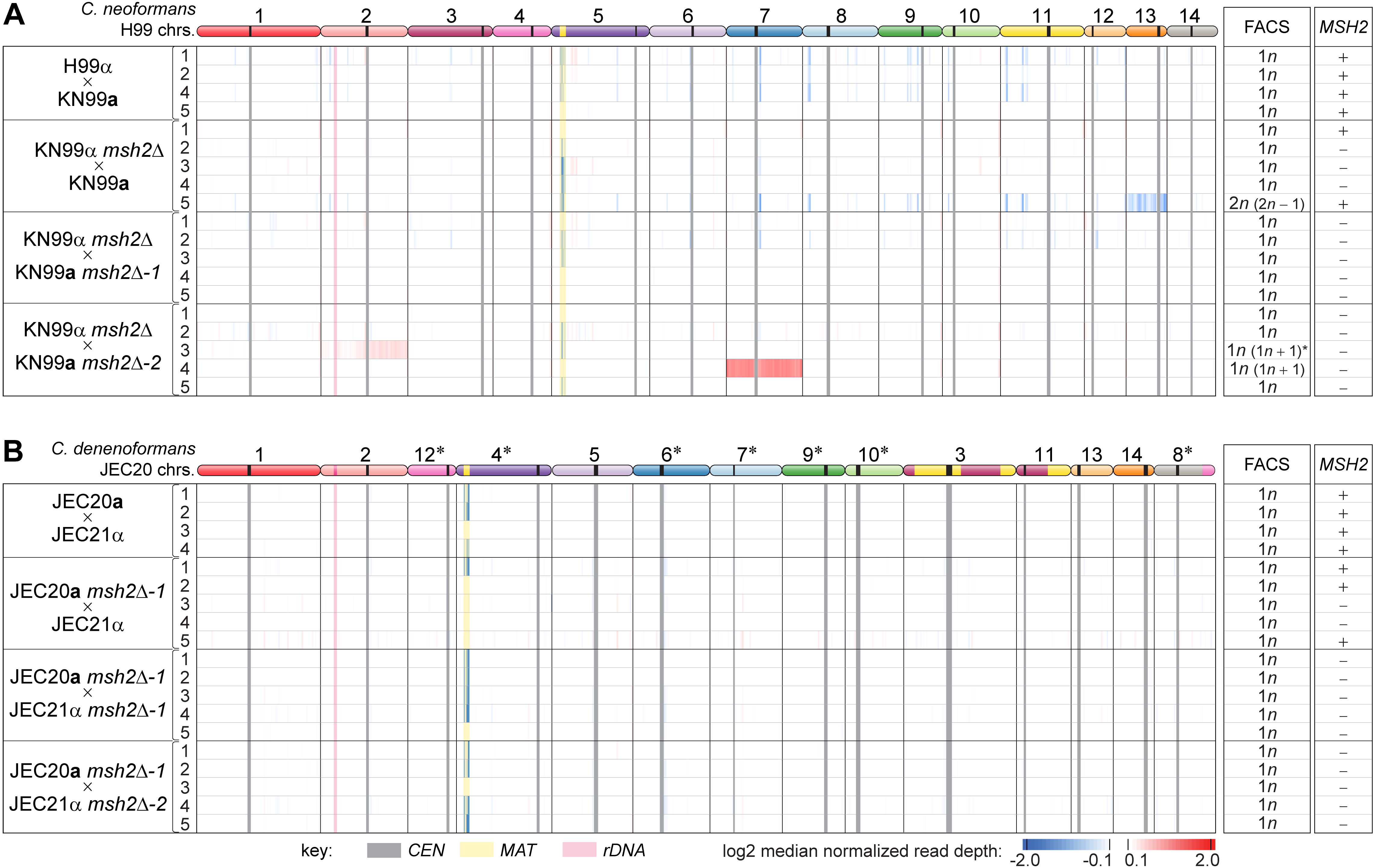

Supplement: S12 Fig — Read depth (binned in 1-kb non-overlapping windows) was plotted along each chromosome of C. neoformans H99 (A) and C. deneoformans JEC21 (B) to screen for chromosome aneuploidy. For each sequenced strain, read depth was normalized to the median read depth for that strain, log2-transformed, and plotted as a heat map in IGV viewer. Ploidy was also measured by FACS and the results indicate that progeny #5 of KN99α msh2Δ × KN99a is mostly diploid except for chromosome 13 (2n -1), and progeny #3 and #4 of KN99α msh2Δ × KN99a msh2Δ-2 have gained additional copies of chromosomes 2 and 7, respectively (1n + 1). The asterisk indicates that the biased sequence coverage observed along Chr2 of progeny 3 from the KN99α msh2Δ × KN99a msh2Δ-2 cross might be due to biochemical effects related to library preparation or sequencing. (TIF) [file pgen.1008871.s022.tif]

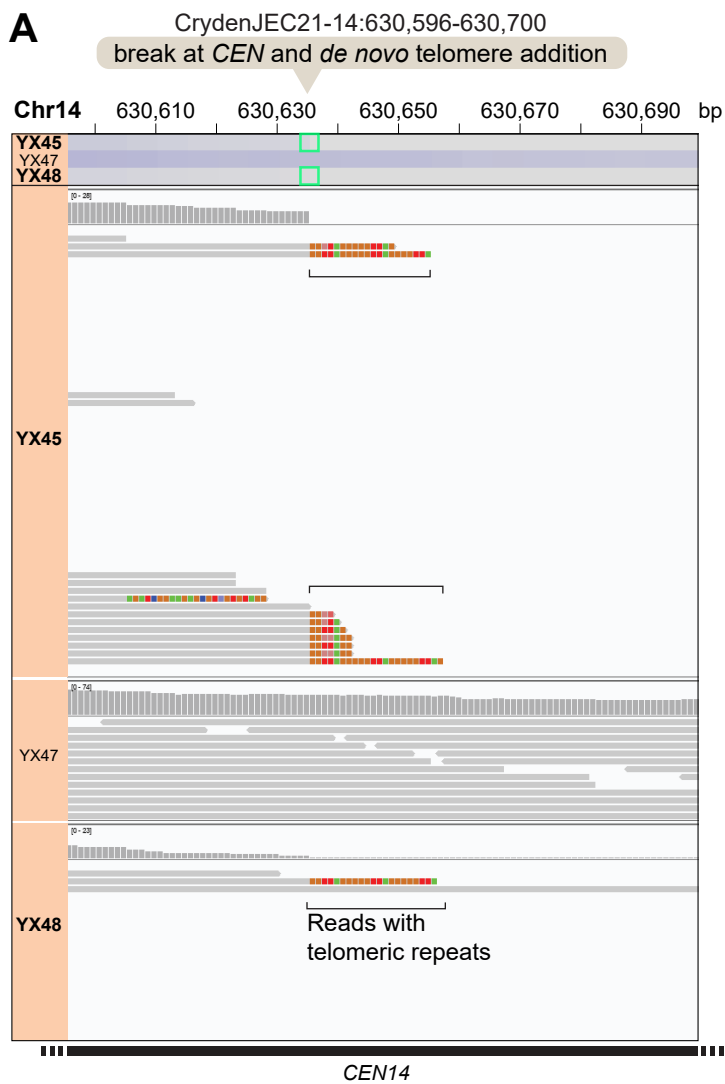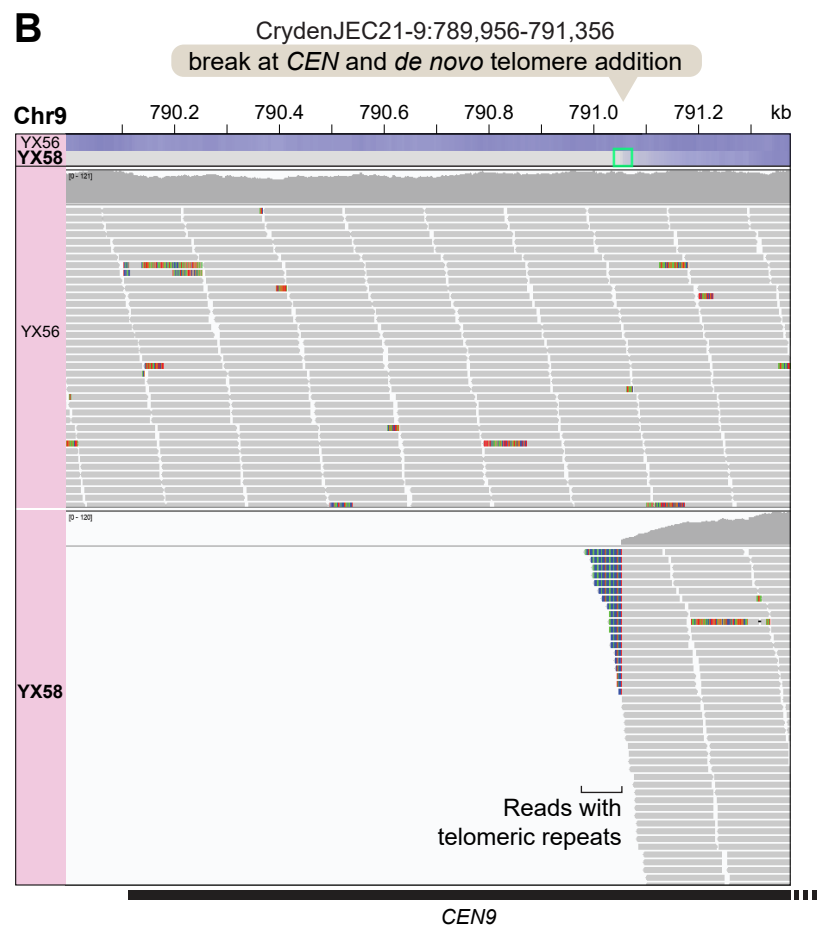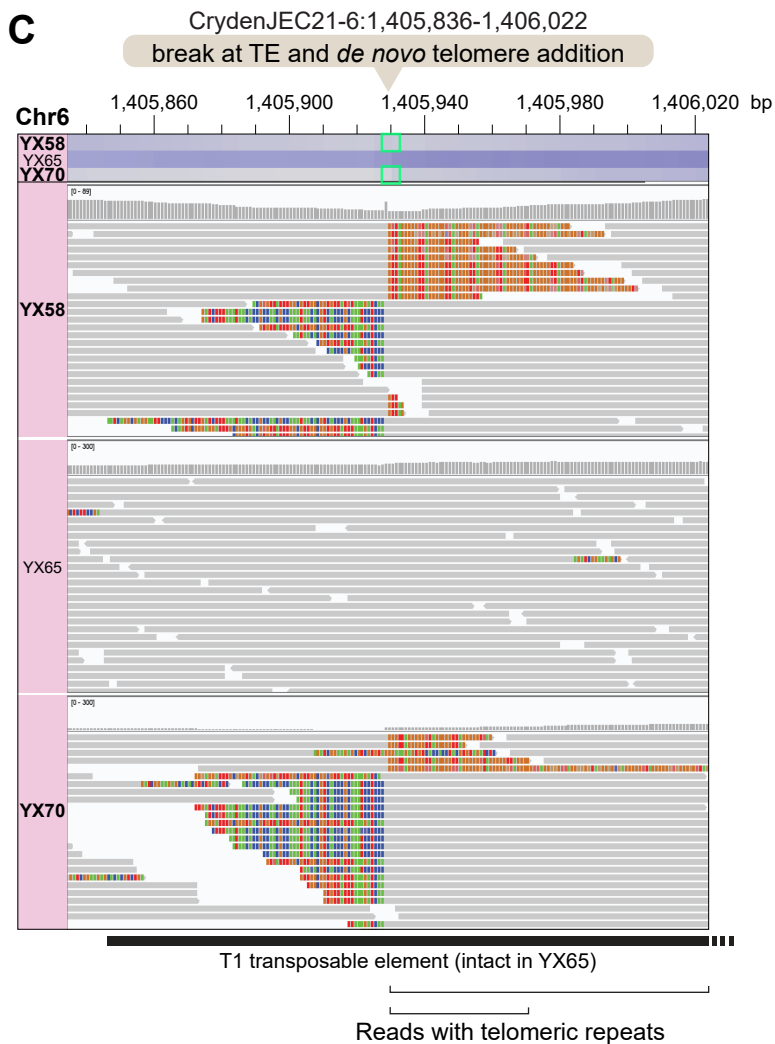

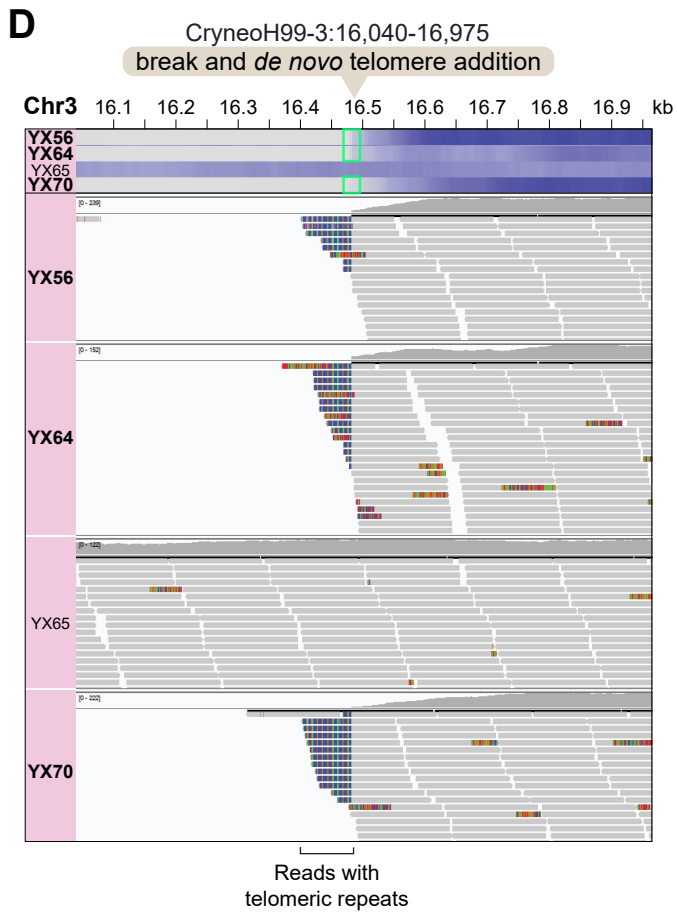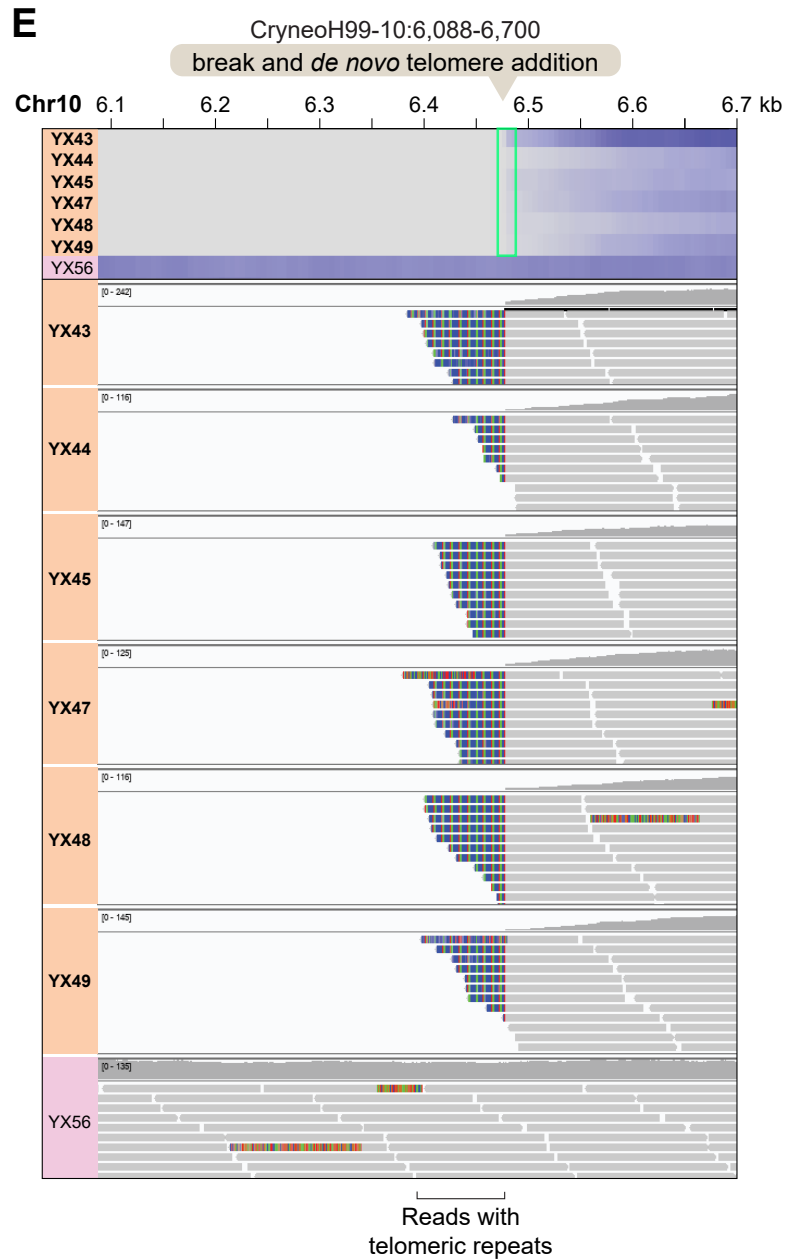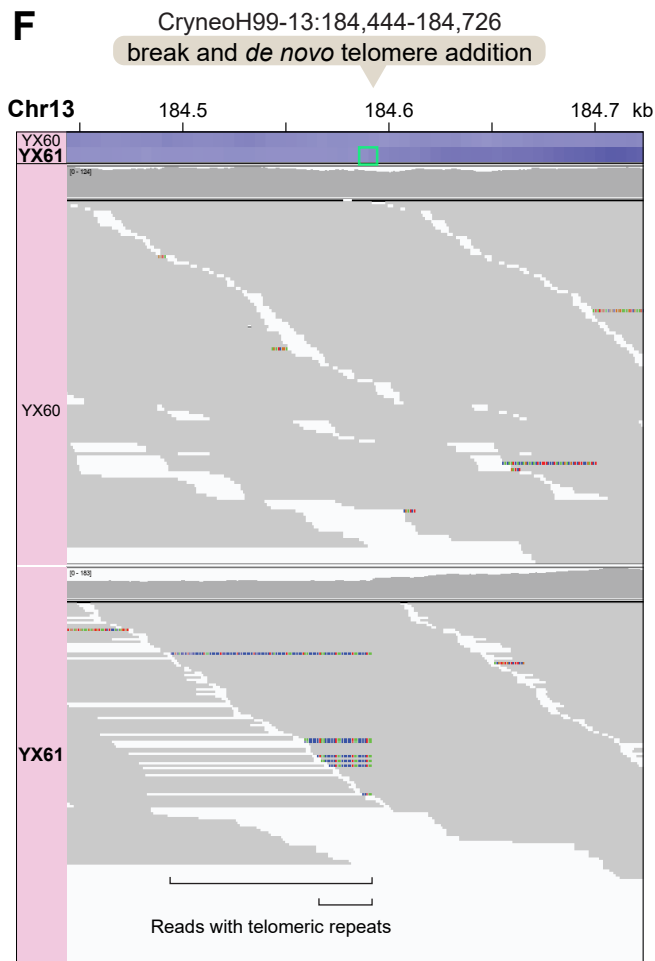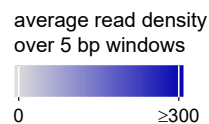

Supplement: S13 Fig — Breakpoints were detected in different chromosomal locations (see S7 Table for details), including CEN14 and CEN9 of JEC21α (A and B), a T1 transposable element located at the end of Chr6 of JEC21α (C), or in other genomic locations (D–F). Each panel shows the result of read mapping for one or more progeny that underwent chromosome breakage and healing via de novo telomere addition (strains names in boldface type) and a control strain in which no breaks were detected on the same region (strain names in normal font type). Breakage and de novo telomere addition was inferred, respectively, by abrupt changes in read coverage (depicted as bars on the top and colored as shown in the key) and by the presence of reads with telomeric repeats at the breakpoints. When two copies of the same chromosome are present, only a subset of reads are expected to contain telomeric repeats (as shown e.g. in panel F). (PDF) [file pgen.1008871.s023.pdf]

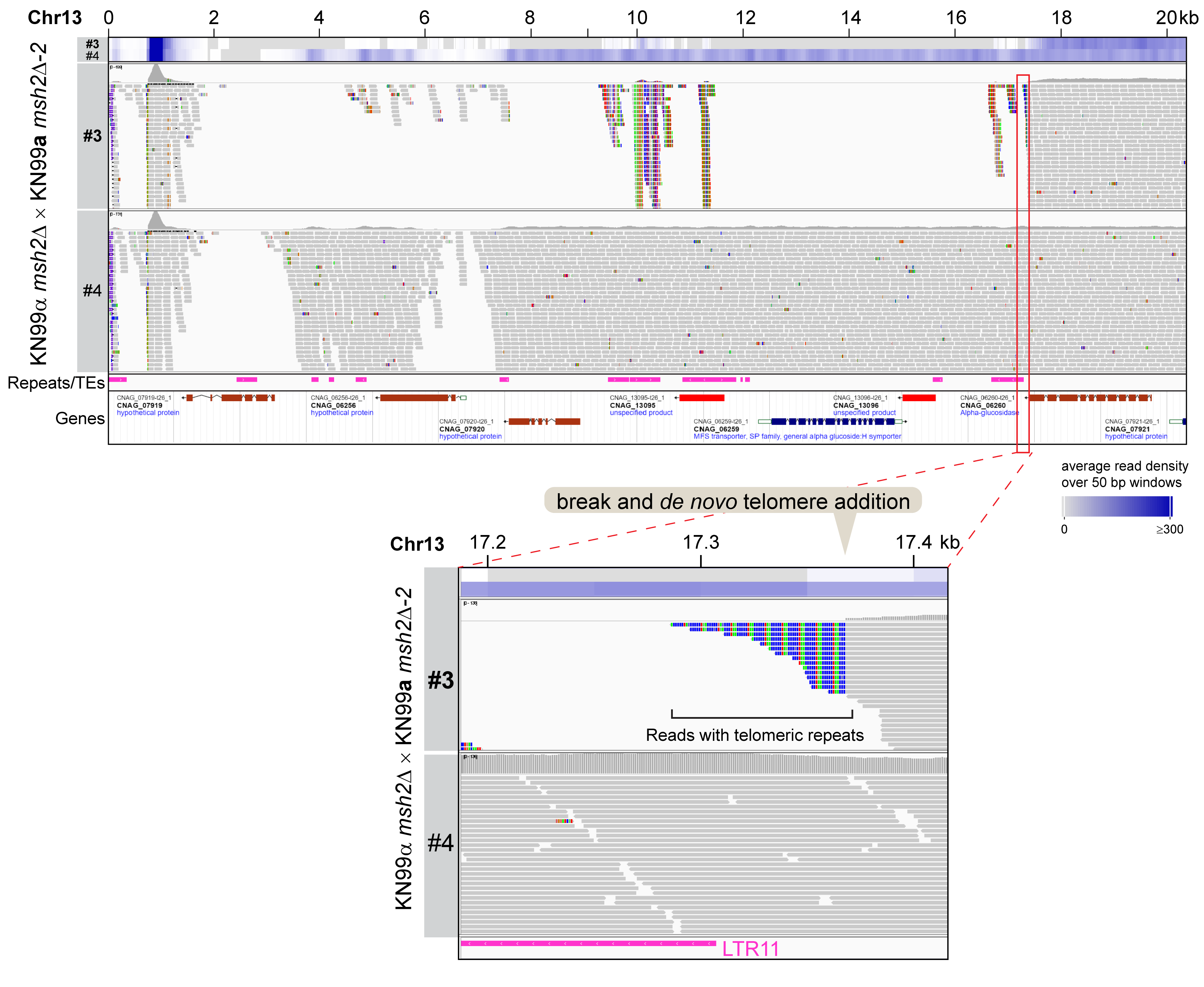

Supplement: S14 Fig — Breakage and de novo telomere addition was inferred, respectively, by abrupt changes in read coverage (depicted as bars on the top and colored as shown in the key) and by the presence of reads with telomeric repeats at the breakpoints. Such events were not detected in any of the 19 C. deneoformans sequenced intraspecific progeny and were found in only 1 progeny (#3 of KN99α msh2Δ × KN99a msh2Δ-2) out of 19 progeny derived from the C. neoformans intraspecific crosses. In this strain, a region of ~17 kb, which contained a few putative genes and predicted transposable elements, was deleted from the 5’ end of chromosome 13. (TIF) [file pgen.1008871.s024.tif]
