# Supplementary material for: Factors enforcing the species boundary between the human pathogens Cryptococcus neoformans and Cryptococcus deneoformans
Source: PLoS Genet. 2021 Jan 19;17(1):e1008871. doi: 10.1371/journal.pgen.1008871 (PMC7846113; doi:10.1371/journal.pgen.1008871)
Supplement: S9 Fig — (A) H99α x JEC20a. (B) H99α x JEC20a msh2Δ-1 (C) KN99α msh2Δ x JEC20a (D) H99α msh2Δ x JEC20a msh2Δ-1. For each progeny, read-depth plots (normalized to the genome-wide average coverage) are colored according to each parental species contribution as shown in the key on the top right, and a schematic representation of the inferred karyotype is depicted on the right. Homeologous chromosomes are color coded based on the H99 reference (see S8 Fig for details) and asterisks in JEC21α indicate chromosomes in reverse-complement orientation. Red arrowheads mark recombination breakpoints between homeologous chromosomes and/or loss of heterozygosity (also highlighted by red boxes in the karyotype panels). Where detected, the breakpoints of additional recombination events within the same chromosome are indicated by light blue arrowheads. Circular black labels: (a) marks changes in ploidy in a subset of the population of cells that were sequenced; (b) marks chromosome breakage events repaired by de novo telomere addition (see S13 Fig for details); (c) indicates recombination events next to the MAT locus; (d) marks a break at the rDNA locus; (e) marks a complex chromosomal aberration that cannot be explained by simple rearrangements and required further investigation. (PDF) [file pgen.1008871.s019.pdf]

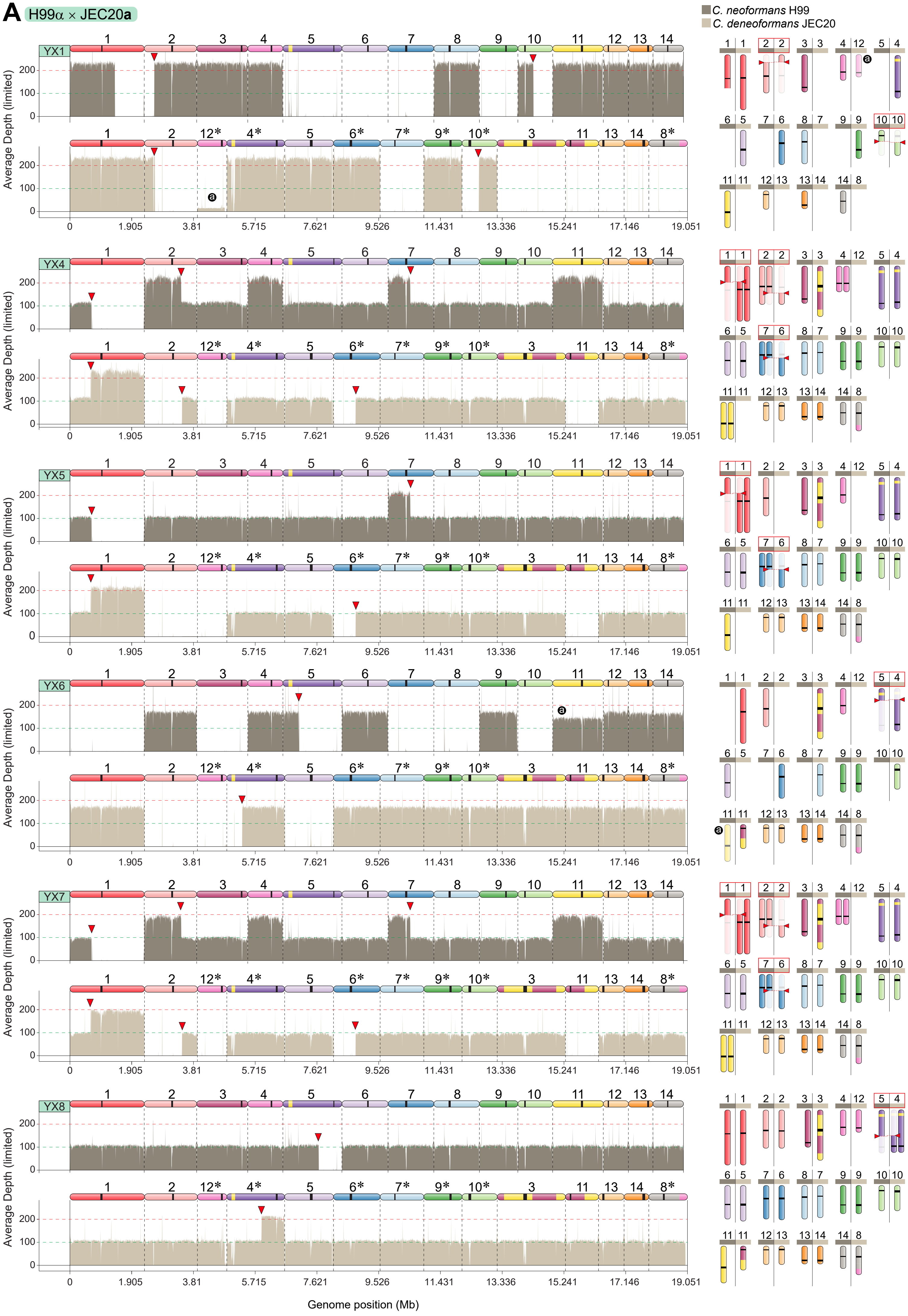
A
H99α × JEC20**a**
C. neoformans H99
C. deneoformans JEC20
YX1
YX4
YX5
YX6
YX7
YX8
1 2 3 4 5 6 7 8 9 10 11 12 13 14
1 2 12* 4* 5 6* 7* 9* 10* 3 11 13 14 8*
Average Depth (limited)
0 100 200
0 1.905 3.81 5.715 7.621 9.526 11.431 13.336 15.241 17.146 19.051
Genome position (Mb)

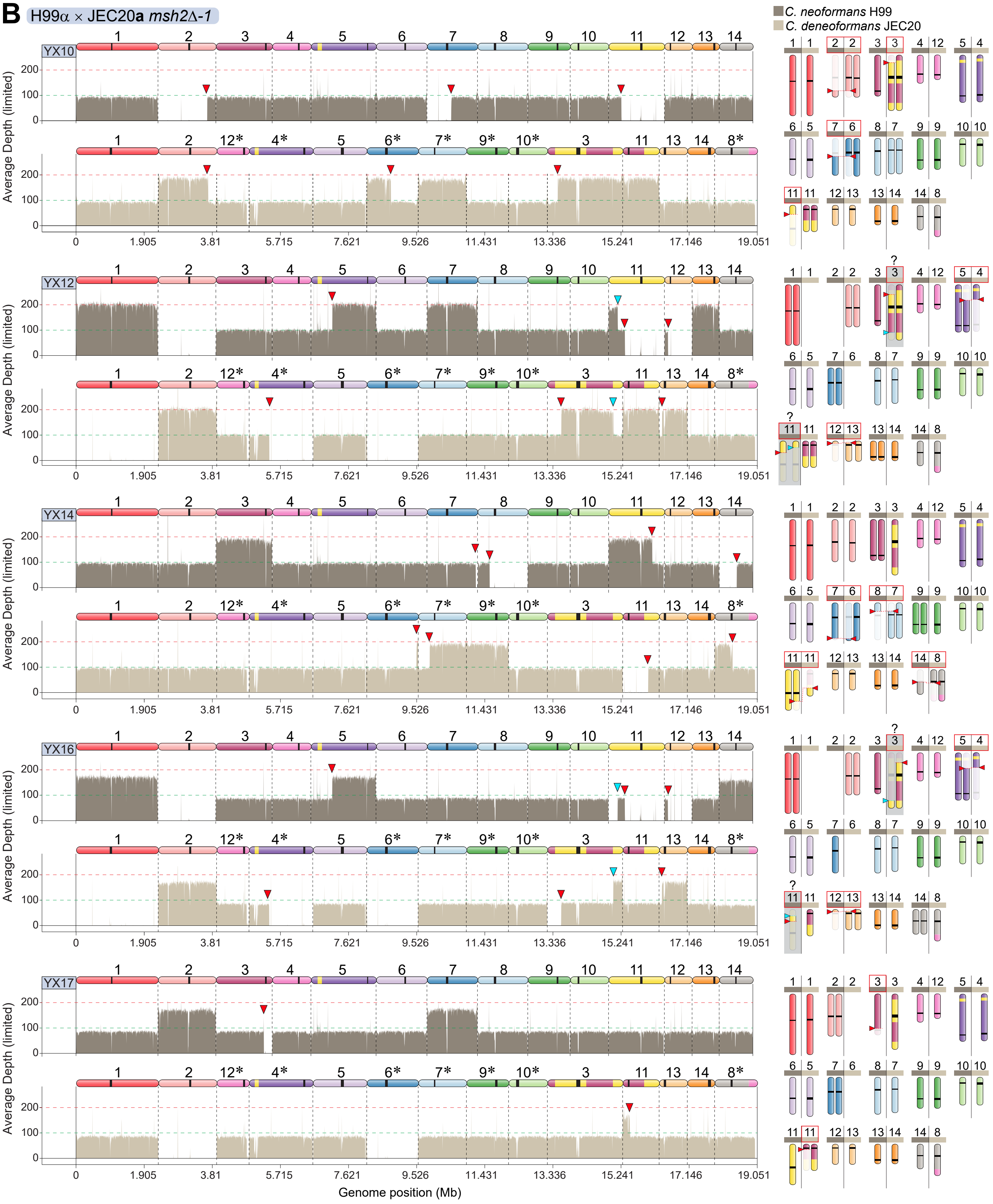
B
H99α × JEC20a msh2Δ-1
C. neoformans H99
C. deneoformans JEC20
YX10
YX12
YX14
YX16
YX17
1 2 3 4 5 6 7 8 9 10 11 12 13 14
1 2 12* 4* 5 6* 7* 9* 10* 3 11 13 14 8*
Average Depth (limited)
0 100 200
0 1.905 3.81 5.715 7.621 9.526 11.431 13.336 15.241 17.146 19.051
Genome position (Mb)

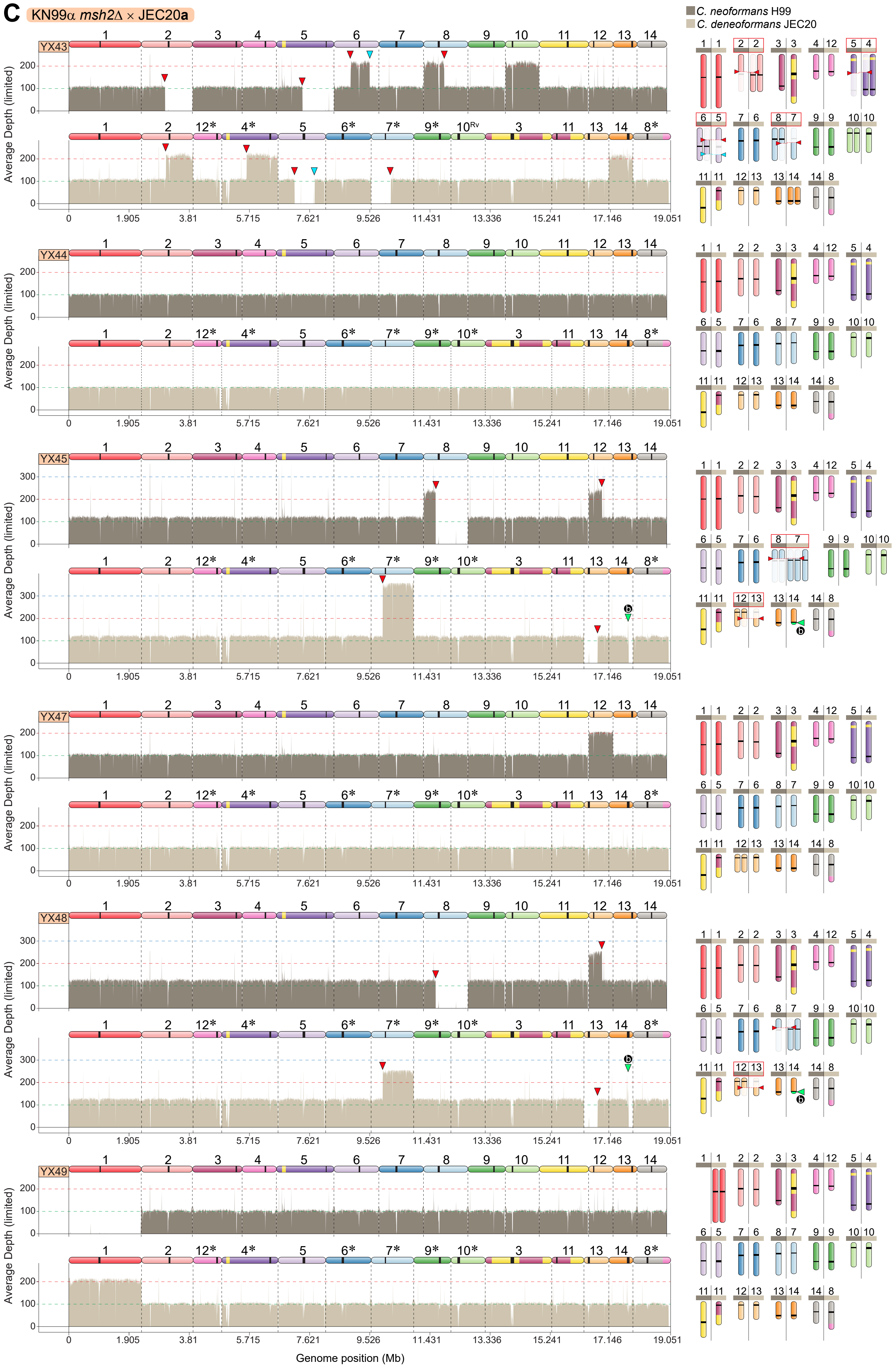
C
KN99α msh2Δ × JEC20a
C. neoformans H99
C. deneoformans JEC20
YX43
YX44
YX45
YX47
YX48
YX49
1 2 3 4 5 6 7 8 9 10 11 12 13 14
1 2 12* 4* 5 6* 7* 9* 10* 3 11 13 14 8*
10Rv
Average Depth (limited)
0 100 200 300
0 1.905 3.81 5.715 7.621 9.526 11.431 13.336 15.241 17.146 19.051
Genome position (Mb)

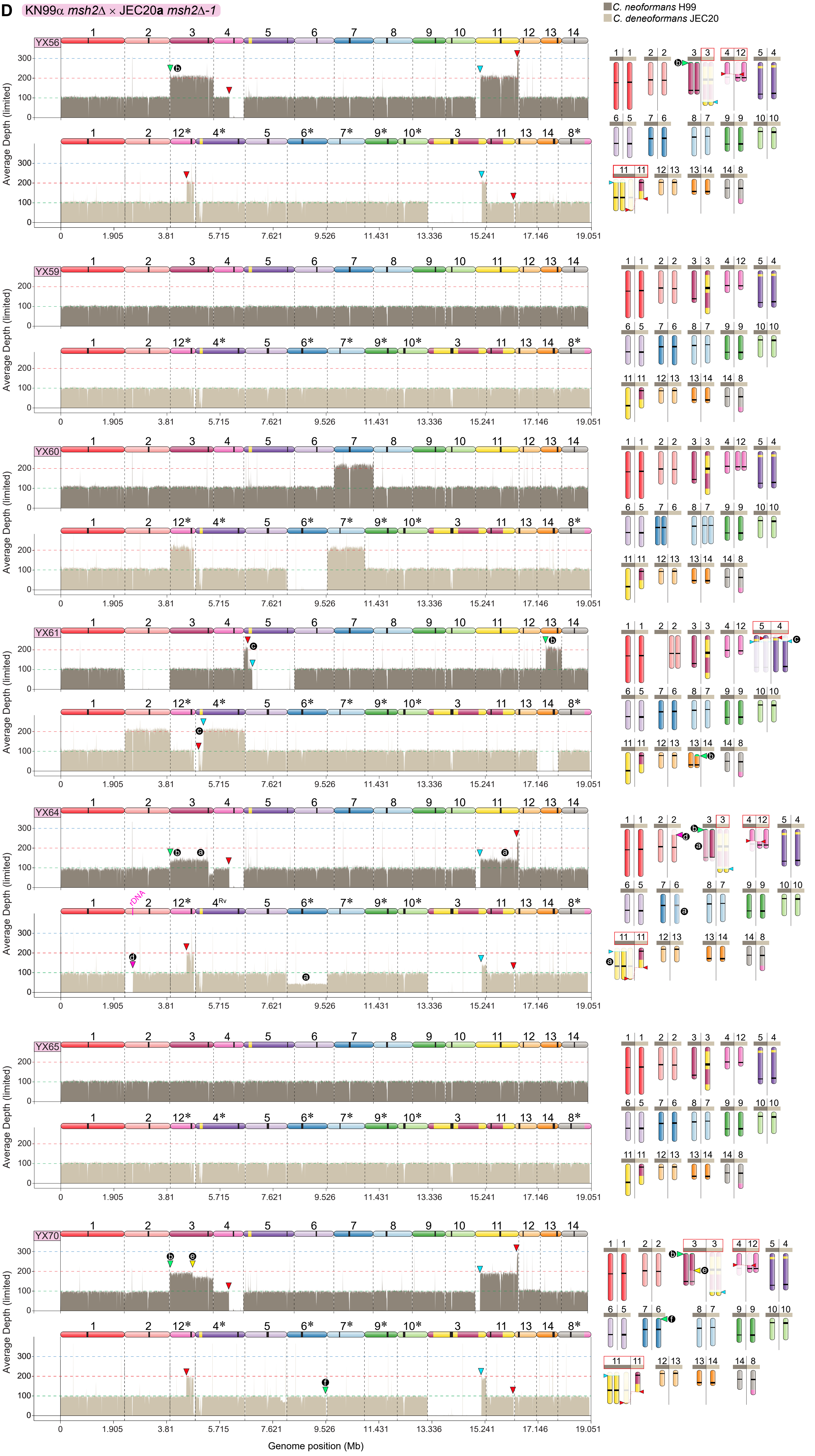

D
KN99α msh2Δ × JEC20a msh2Δ-1
C. neoformans H99
C. deneoformans JEC20
YX56
YX59
YX60
YX61
YX64
YX65
YX70
Average Depth (limited)
rDNA
Genome position (Mb)
0
1.905
3.81
5.715
7.621
9.526
11.431
13.336
15.241
17.146
19.051
