# Supplementary material for: Factors enforcing the species boundary between the human pathogens Cryptococcus neoformans and Cryptococcus deneoformans
Source: PLoS Genet. 2021 Jan 19;17(1):e1008871. doi: 10.1371/journal.pgen.1008871 (PMC7846113; doi:10.1371/journal.pgen.1008871)
Supplement: Attachment — Submitted filename: Response to Reviewers.docx [file pgen.1008871.s025.docx]

**[Responses to reviewers’ comments]**

**REVIEWER COMMENTS**

**Reviewer #1**: In this study the authors performed crosses between two species of Cryptococcus (C. neoformans and C. deneoformans, ~15% divergent), and then analyzed progeny that were derived from wild-type or mismatch repair defective (msh2 null, unlateral or bilateral) parents. They found that progeny derived from msh2null bilateral crosses showed higher viability and levels of aneuploidy. Recombination events were seen at low frequency in the progeny (1 to 1.5 events per cross), with similar frequencies in wild-type and msh2null crosses. Based on these observations, the authors suggest that the mismatch repair system is not playing a role in limiting meiotic recombination between divergent Cryptococcus species.

In general, the work is well done and clearly explained and shows a role for the mismatch repair factor Msh2 in enforcing species boundaries in Cryptococcus. I have two major comments indicated below.

**Response**: We thank the reviewer for appreciating the quality of our studies.

1. The authors do a nice job describing the previous work in bacteria (Hfr crosses between E. coli and Salmonella typhimurium) and yeast (meiotic recombination involving interspecific hybrid of S. cerevisiae and S. paradoxus) indicating that the mismatch repair system acts to maintain species barriers. However, in addition to these studies, it’s also worth thinking about the following:

Elliott and Jasin (MCB 21:2671) measured I-SceI induced recombination between divergent substrates in mammalian cells. They observed that substrates with 1.5% divergence displayed 10-fold higher levels of recombination in Msh2-/- compared to wild-type cells. In addition, the Msh2-/- recombinants displayed a much higher level of uncorrected tracts, indicating that many recombination events involved the formation of heteroduplex DNA.

Anand et al. (Nature 544:377) examined break-induced replication in yeast using templates that contained different densities of DNA polymorphisms. A key observation of their work was that “When recombination occurs without a protruding nonhomologous 3' tail, the mismatch repair protein Msh2 does not discourage homeologous recombination. However, when the DSB end contains a 3' protruding nonhomologous tail, Msh2 promotes the rejection of mismatched substrates.” In addition, they showed that “Nearly all mismatch correction depends on the proofreading activity of DNA polymerase-δ, although the repair proteins Msh2, Mlh1 and Exo1 influence the extent of correction.”

Peterson et al. (Mol Cell doi: 10.1016/j.molcel.2020.04.009) examined meiotic recombination in F1 hybrid mice. They analyzed events at two recombination hotspots loci that in the hybrid displayed a high (~1%) polymorphism density. Interestingly, they observed no difference in the frequency of crossover events in wild-type and Msh2-/-, but still observed high levels of heteroduplex DNA in Msh2-/-.

I summarized the above studies because they involved systems where a DSB was induced/occurred at a specific site/region and polymorphisms were present that allowed them to monitor the presence of heteroduplex DNA. In addition, the studies illustrate the different outcomes that can be obtained when mismatch repair is ablated. In some cases, mismatch repair had no effect on the overall frequency, but did affect the repair of heteroduplex intermediates. With this said I have the following concerns with the work presented by Priest et al.:

**Response:** We thank the reviewer for sharing details on these insightful studies about the various possible outcomes for recombination in the presence and absence of Msh2 as well as the implications for heteroduplex DNA. In each of the studies referenced by the reviewer as well as other studies in *Saccharomyces* (such as Martini et al. *PLOS Genetics*. 2011), the experimental models used are ideal for detecting recombination and/or assessing heteroduplex DNA (hetDNA). In the study from Elliot and Jasin, the authors either induce DSBs and use a repair substrate with known levels of divergence or assess recombination of an engineered intra-chromosomal recombination system. In the study from Anand et al., the authors utilize a 108-bp donor template with various levels of divergence, with and without a 3’ homologous or non-homologous tail to repair an induced DSB at an HO recognition site. In the study from Peterson et al., the investigators are able to assess recombination and hetDNA by screening tens of thousands of sperm from a limited number of mice at two known recombination sites to fully characterize changes in recombination and patterns of hetDNA. In studies involving *Saccharomyces*, investigators typically apply tetrad dissection to assess the full spectrum of recombination products from a single meiotic event.

In our study, we unfortunately do not have these opportunities because we are assessing the genomes of recombinant progeny derived from random spore dissection. In *Cryptococcus*, random spore dissection yields progeny that are expected to be derived largely or entirely from independent meiotic events; therefore, the analyzed progeny do not represent all possible products from a single meiotic event, but a random sampling of many meiotic events and therefore prevent us from detecting potential evidence of hetDNA using known techniques. Because of this limitation with the dataset used in our study, we cannot detect the products of hetDNA with confidence.

The studies shared by the reviewer do present some interesting outcomes regarding the frequency of recombination events in MMR-proficient and MMR-deficient cells, such as the fact that Peterson and colleagues observed no change in recombination frequencies at 1.8% divergent recombination hotspots in sperm from Msh2-deficient mice compared to wild-type mice, while Elliot and Jasin observed a 10-fold increase in recombination of 1.5% divergent substrates in mouse embryonic stem cells in MMR mutants. The authors of Peterson et al. suggest that these two different outcomes may be due to a higher threshold for the number of mismatches in recombination substrates in meiotic cells relative to mitotic cells. These studies all suggest that there are many mechanisms operating that might lead to small or no increase in homeologous recombination in Msh2-deficient cells. We have included references to these studies within the discussion section of the revised manuscript and discuss their implications for our findings (Lines 563-565).

Future studies in *Cryptococcus* could be dedicated to dissecting spores from single basidia derived from wild-type and *msh2*Δ hybrid crosses (approximating tetrad dissection in *S. cerevisiae* given that previous studies have provided evidence for a single meiotic event per basidum), and characterizing the full spectrum of recombination events that can be produced from a single meiosis through whole-genome sequencing. This analysis could provide great insight into the frequency and tract length of unrepaired hetDNA that forms during recombination. However, these studies are outside the scope of this project, but will be of great interest in the future.

**Lines 563-565**: Conversely, studies in other models have observed instances where loss of Msh2 did not lead to increased recombination frequencies between substrates with as little as 1% sequence divergence, up to 25% divergence [74–76]

A. Crosses between the two species yielded very low numbers of events (1 to 1.5 per cross), and as the authors stated on line 479, it is “difficult to determine if these recombination events occurred during meiosis or subsequent mitotic growth during the culturing required for whole genome sequencing.”

**Response**: We appreciate the reviewer’s concern about this statement. A previous study from our lab (Li et al. 2012. *G3.*) showed that loss of heterozygosity can occur during mitotic growth of *C. neoformans* x *C. deneoformans* hybrids, due to either loss of whole or partial chromosomes, which could lead to the generation of novel, recombinant chromosomes. Two other studies (Sun et al. 2014. *PLOS Genetics*.; Vogan et al. 2013. *PLOS One*) have also shown results that suggest mitotic recombination can even occur during the mating process itself. Following these findings and due to the high level of aneuploidy and the subsequent rounds of mitotic division these hybrid progeny will have undergone during the necessary culture growth prior to sequencing, it is not inconceivable that recombination may have occurred between non-sister homologous chromosomes during this mitotic growth. Thus, we cannot conclude whether instances of LOH and recombination observed in the whole-genome sequencing data occurred during meiosis, mitotic recombination during mating, or subsequent mitotic growth required to conduct whole-genome sequencing. Details of these studies have been incorporated into the discussion to provide more context and we have adjusted the wording of this statement as well.

**Lines 539-542**: One caveat to note regarding the recombination events identified through WGS, is that *C. neoformans* x *C. deneoformans* hybrids have been known to experience loss of heterozygosity during mitotic growth [45], and results from other previous studies also suggest that mitotic recombination can occur during mating itself [47,69].

B. Their analysis did not map the exact breakpoints or determine whether there was a significant polymorphism density in the regions undergoing recombination, or if there was any evidence of uncorrected repair tracts. It was intriguing that the authors observed a fair number of de novo telomere addition events in msh2 bilateral crosses, perhaps indicative of an increase in breakage under these conditions. Because it’s not clear where the breakpoints occurred, it’s hard to determine if the breaks occurred in repetitive regions where one might not expect to see effects of mismatch repair. These issues make it difficult for me conclude that the authors can provide mechanistic evidence for factors enforcing the species boundary between different Cryptococcus species.

**Response**: This is an insightful suggestion! Accordingly, we have conducted additional analysis to address this point and we hope the reviewer finds our efforts satisfactory. Our new analysis shows that recombination events occurred in regions with average levels of polymorphism densities and that recombination events were not more likely to occur at repetitive elements (Figure 6). Recombination breakpoints are also provided in Table S6.

The observed *de novo* telomere additions occurred in a variety of genomic contexts, including at the centromeres (see supplemental Figure 13 panels A and B), which are characterized by repetitive retrotransposable elements in *Cryptococcus*, at transposable elements located elsewhere in the genome (see supplemental Figure 13 panel C), and at non-repetitive regions in the genome (see supplemental Figure 13 panels D, E, and F). Of the 15 instances of *de novo* telomere repeat addition observed in the hybrid progeny genomes, only 5 of these events occurred at repetitive elements (centromeres or transposons), suggesting that these events are not more likely to occur at repetitive elements. These instances of *de novo* telomeric repeat addition do not likely contribute to the maintenance of the species boundary between *C. neoformans* and *C. deneoformans*, but were a result that we felt was important to report.

Although the reviewer correctly points out that chromosomal breakage is more likely to occur in MMR mutants in other species, we hypothesize that these events of breakage followed by *de novo* telomere addition are much more likely to occur in the context of a diploid *Cryptococcus* strain, because loss of the remainder of the chromosome will not be deleterious. Supporting this hypothesis, we found only a single instance of chromosomal breakage followed by *de novo* telomeric repeat addition in intraspecific progeny derived from a bilateral *msh2*Δ *C. neoformans* cross (see Supplemental Figure 14).

2. The authors report low rates of loss of heterozygosity and high rates of aneuploidy in hybrid progeny from msh2null mutant crosses. This is an interesting observation but, at least for this reviewer, not a lot of mechanistic insights were provided. It would be interesting to perform crosses within the same species (C. neoformans x C. neoformans, C. deneoformans x C. deneoformans), analyzing progeny derived from both wild-type and msh2null isolates. This would provide information on whether some of the phenotypes that were observed were the result of sequence divergence or other activities promoted by Msh2.

**Response**: This is an excellent point and we have now generated congenic mating partners lacking *MSH2* in both parental species. We have conducted the suggested intraspecific crosses involving wild-type and *msh2*Δ parents and assessed the progeny produced by these crosses. Of the 38 total progeny that we have whole-genome sequence as well as FACS analysis data for, only three progeny from all wild-type, unilateral *msh2*Δ, and bilateral *msh2*Δ *C. neoformans* x *C. neoformans* displayed aneuploidy: one progeny had a ploidy of 2*n*-1 and two had a ploidy of 1*n*+1 (See Figure S12). No progeny derived from any of the wild-type, unilateral *msh2*Δ, and bilateral *msh2*Δ *C. deneoformans* x *C. deneoformans* crosses displayed any aneuploidy (See Figure S12). Because high levels of aneuploidy are a known characteristic of *C. neoformans* x *C. deneoformans* hybrids, these findings suggest that the increased levels of loss of heterozygosity observed in hybrid progeny derived from crosses involving two parents lacking Msh2 are related specifically to the loss of Msh2 in the context of hybrids.

Other comments

1. Line 70: Define what you mean by “transgressive. A violation of accepted dogma?

**Response**: This is a great question, because transgressive does have several different meanings. In this study, we are using the word transgressive as it is traditionally used in the field of genetics: referring to a characteristic not observed in either parent or a characteristic that is not intermediate to the characteristics observed in either parent, i.e. a characteristic outside of the expected range, or an extreme phenotype. For instance, in the manuscript, the ability to produce mating structures on YPD medium is a transgressive phenotype because neither parent is capable of producing sexual reproduction structures on YPD medium. We have also clarified this in the introduction section as well.

**Lines 162-165**: *C. neoformans* x *C. deneoformans* hybrids also have unstable karyotypes, can be self-fertile, and display phenotypes intermediate of either parent, although instances of transgressive phenotypes (i.e. phenotypes that fall outside of the range between either parental phenotype) and hybrid vigor have been observed [41,43–45].

2. Lines 109-121. The authors provide many details about how many hybrids exist in nature, but they don’t address how often they are fertile. This paragraph is nicely written, but feels like a distraction from the topic they are addressing (factors that enforce species boundaries).

**Response:** The reviewer makes a great point, and we have incorporated a statement in the manuscript to clarify the infertility associated with many hybrids in nature. We have also revised the paragraph to make it more concise.

**Lines 107-117:** Despite the numerous pre- and post-zygotic species barriers and the robust fitness defects associated with hybrids, hybridization occurs at an appreciable frequency. In an exciting recent study, researchers witnessed the origin and monitored the evolution of a novel finch species that arose through a hybridization event in the Galapagos Islands [12]. Hybridization has impacted recent human evolution as well, with several modern human populations having introgressed genomic regions from Neanderthals or Denisovans [13]. Ligers, the hybrid progeny of a male lion and female tiger, and mules, the hybrid progeny of a male donkey and female horse, are examples of hybrids that normally only occur through human intervention. Ligers are bred for their size, as they are larger than either parent (an instance of hybrid vigor), while mules are bred for their endurance, docile demeanor, and intelligence [14]. It is important to note however, that interspecific hybrids are often sterile [15].

3. Line 197. It would be worth explaining here what the targets are in the rapamycin +FK506 (FRR1 is a common target) and 5-FOA (URA3 or URA5) assays that lead to resistance to these drugs. This would give the reader an immediate context in terms of the kinds of mutations that can be identified.

**Response**: This is a great suggestion! We have revised our manuscript accordingly and provided additional relevant details in the text.

**Lines 200-203**: Resistance to the combination of rapamycin and FK506 is mediated by mutations in their common target, FKBP12, which is encoded by the gene *FRR1*, while resistance to 5-FOA arises following loss-of-function mutations in *URA5* or *URA3*, genes encoding enzymes involved in the *de novo* pyrimidine biosynthesis pathway.

4. The detailed analysis of the msh2null-1 mutant feels like a distraction. It’s somewhat interesting that the authors could track down why msh2null-1 failed to produce 5-FOA resistant isolates, but the extended analysis of the mutant does not provide much to the overall story. I see no reason why it can’t be presented in the Materials and Methods or supplement, rather than being emphasized in the Results and Discussion.

**Response:** We concur with the reviewer and have shortened the description of these findings in the results section and moved the figure panels into a supplemental figure (see Figure S2).

5. Line 253 and elsewhere. It would be valuable in the text to provide some of the statistical analysis presented in the Figures that indicated that the differences seen between wild-type and msh2null were significant.

**Response**: We thank the reviewer for this constructive feedback and have adjusted the manuscript to include additional statistical analyses. The revised manuscript now includes additional statistical analyses of our results regarding the production of bald basidia in hybrid genetic crosses, the germination frequencies of both hybrid and intra-specific progeny, and the ability of hybrid progeny to produce sexual reproduction structures on YPD medium. See Figure 2, 3, and S3, and Tables S1, S2, and S3.

6. Line 491. Typo-do you mean 15% divergent sequences?

**Response**: We apologize for this error and have revised the text accordingly.

**Reviewer #2**: Priest et al. aim to identify the role of the gene MSH2 in hybrid sterility in the pathogenic fungi Cryptococcus. Previous work in Saccharomyces has shown that anti-recombination due to sequence divergence is responsible for sterility between Saccharomyces species. Knocking out MSH2 can rescue part of this sterility by increasing the number of meiotic events between divergent sequence and thus promoting proper chromosome segregation. Here, the researchers show that MSH2 does function in DNA MMR in Cryptococcus similar to other organisms, but does not increase successful recombination in hybrids. This is at odds with their results that germination frequency is greatly increased, and it is unclear what mechanism is leading to higher viability. They also demonstrate other interesting results, like high rates of diploidy in the viable hybrids, and potentially an ability for hybrids to self fertilize. They thus show support that MSH2 has diverged in function in Cryptococcus, surprising as its role in anti-recombination is conserved from bacteria to eukaryotes.
I think the topic of hybridization in fungal pathogens is really interesting, and a better understanding of the mechanisms and phenotypes associated with hybrid viability is needed. I particularly enjoyed the Discussion section where this was addressed. I have several larger questions and comments, below, and some clarifying questions I think would lead to a more clear manuscript.

**Response**: We thank the reviewer for appreciating the quality of our studies.

Throughout the manuscript there is a lot of reference to phenotypes “unique to hybrid progeny,” but it’s unclear if these are unique to hybrid progeny because non-hybrid crosses are not analyzed (or at least not discussed). For example, is the ability to undergo sexual reproduction after 3 weeks under abnormal conditions unique to msh2 hybrids? What do wild-type hybrid and non-hybrid cross controls look like? What are the difference between hybrids and non-hybrids under the same conditions after this time period?

**Response**: This is an excellent question and we have added clarification in the text as well as additional data to the revised manuscript. The ability to produce sexual reproduction structures specifically on YPD medium after 3 weeks is a phenotype that has not been previously observed in *C. neoformans* or *C. deneoformans* strains, nor has it been observed in any matings or hybrids of these species. Additional analysis has been conducted to quantify the number of hybrid progeny capable of displaying this phenotypes and appropriate controls have been included to show that this phenotype is specific to the hybrid progeny of this study and not observed in parental strains, or other common laboratory *C. neoformans* or *C. deneoformans* strains. However, there was one exception: the *C. deneoformans* hyperfilamentous strain XL280 and strains derived from XL280 were able to produce extremely sparse hyphae under these YPD conditions. However, this phenotype of hyphal production on YPD has not been reported in any other strains previously.

**Lines 288-299:** Continued incubation and additional microscopic analysis revealed that after approximately three weeks of incubation, a number of these progeny produced basidia and sparse basidiospores under conditions previously not known to support sexual reproduction for any *Cryptococcus* species or strains (rich medium, incubated in the light in sealed plates) (Fig 3). This phenotype was measured across all hybrid progeny, parental strains, and standard laboratory reference strains. Although there was no significant difference between the ability to produce hyphae on YPD between the progeny from the different types of hybrid crosses (one-way ANOVA), progeny from bilateral *msh2*Δ hybrid crosses tended to be able to produce hyphae more often on average than progeny from wild-type or unilateral *msh2*Δ hybrid crosses (45% compared to 25% or 19%, respectively) (Fig 3B and S3 Table). Interestingly, no parental strains, progeny from intraspecific crosses, or common laboratory reference strains were able to produce hyphae on YPD except for XL280α, a hyper-filamentous *C. deneoformans* strain [55].

Statistics are needed to identify if differences are significant.

**Response**: We thank the reviewer for this constructive critique and have included additional statistical analyses to determine if this ability to produce hyphal filaments on YPD in hybrid progeny was significantly different across the different types of hybrid crosses. The revised manuscript also now includes statistical analyses of our results regarding the production of bald basidia in hybrid genetic crosses, and the germination frequencies of both hybrid and intra-specific progeny. See Figure 2, 3, S3, and Tables S1, S2, and S3.

Similarly, the observation of repairs of double strand breaks with telomeric repeat sequences (Lines 341-345) - is this unique to hybrid progeny with msh2 or does this happen in msh2 knockouts in non-hybrid progeny as well?

**Response**: We hypothesize that this phenomenon of *de novo* telomere addition might be much more frequent in hybrid progeny due to the high levels of aneuploidy in these strains. This is because loss of large chromosomal regions is not likely to be deleterious in the context of a diploid background, unlike the typically haploid *C. neoformans* and *C. deneoformans* strains and their intraspecific progeny. Supporting this, in all instances of *de novo* telomeric repeat addition in the hybrid progeny that were observed, another full-length copy of the homologous chromosome was also present, thus sheltering any deleterious effects of partial chromosome loss in the chromosome that underwent *de novo* telomeric repeat addition. Because aneuploidy is much less frequent in non-hybrid progeny, it is less likely that progeny with chromosomes that have undergone breakage and been healed by *de novo* telomeric repeat addition would be viable. In our analysis of progeny from wildtype, unilateral *msh2*Δ, and bilateral *msh2*Δ *C. neoformans* and *C. deneoformans* intraspecific crosses, we only observed one *de novo* telomere addition event that occurred at the distal end of a chromosome, resulting in the loss of only a small number of putative genes with unknown function (See Figure S14).

I’m not sure I agree with the statement that loss of Msh2 did not relax species boundaries in Cryptococcus. It seems from their results of germination frequency, that MSH2 does indeed play a role in maintaining species boundaries (when msh2 is deleted in both species, germination increases), in line with results from other organisms. The statement that alternative pathways limit recombination is also unclear, as only MSH2 was tested in this capacity and the MMR pathway still appears to be functioning in many aspects of chromosome segregation/meiosis.

**Response**: We agree with the reviewer and have revised the narrative of the manuscript to more clearly indicate that loss of Msh2 did relax the species boundary, as evidenced by the higher viability of progeny derived from crosses between parents lacking Msh2, but did not relax the species boundary in all expected ways, such as increasing meiotic recombination.

Could you quantify the aneuploidy you see? It would be helpful to see proportions of crosses with aneuploidy across non-hybrids, non-hybrid msh2, msh2 knockouts in wild type hybrids, unilateral and bilateral msh2 hybrids. Is the aneuploidy random or are there particular chromosomes that are lost or gained from one species?

**Response**: We have quantified the aneuploidy observed in the hybrid progeny assessed in this study (Table S4 and Fig S10). In addition, we have conducted additional intra-specific crosses and derived progeny from wild-type, unilateral *msh2*Δ, and bilateral *msh2*Δ *C. neoformans* and *C. deneoformans* intraspecific crosses to address the reviewer’s question. A total of 38 intra-specific progeny were sequenced at the whole-genome level. Of the 38, only one progeny was highly aneuploid (KN99α *msh2*Δ x KN99**a** progeny #5 was shown to be 2N-1 through a combination of whole-genome sequencing and FACS analysis). Aside from this nearly diploid progeny, only two other progeny appeared to be aneuploid for a single chromosome (KN99α *msh2*Δ x KN99**a** *msh2*Δ progeny #3 and #4 were assessed to be 1N+1). The remaining 35 progeny from the various intraspecific wild-type, unilateral *msh2*Δ, and bilateral *msh2*Δ crosses were haploid as characterized by the combination of WGS and FACS analysis. See Figures S6 and S12.

Is recombination required for successful completion of meiosis in Cryptococcus?

**Response**: Similar to their roles in *S. cerevisiae*, Spo11, the initiator of double-strand DNA breaks during meiosis, and Dmc1, the meiosis-specific recombinase, are required for efficient meiosis in *Cryptococcus*. In studies of *Cryptococcus* mutants lacking Spo11, *Cryptococcus* matings were unable to produce viable meiotic progeny (Feretzaki and Heitman. *PLOS Genetics*. 2013.). In studies of mutants lacking Dmc1, *Cryptococcus* matings were unable to efficiently undergo sexual reproduction and were only able to produce basidia with stunted, short, or intermediate-length spore chains, which is very different from the long basidiospore chains that emerge from normal basidia; germination frequencies of progeny derived from *dmc1*Δ mutants is also reduced to 1.5% (Lin et al. *Nature*. 2005.).

What is the expected meiotic recombination frequency?

**Response**: A previous study in *C. deneoformans* (Roth et al. 2018. *Genetics*.) found that the meiotic recombination frequencies in *C. deneoformans* progeny derived from bisexual crosses was ~1.27 per chromosome per offspring and was ~0.97 per chromosome per progeny derived from unisexual crosses. The recombination frequencies observed in previous studies of *C. neoformans* x *C. deneoformans* hybrid progeny were calculated as ~85 Kb/cM, which is approximately 6 to 7 times lower than those in the intraspecific crosses (Sun and Xu. *Genetics*. 2007.). The recombination frequencies from Roth et al. and Sun and Xu have been included in the discussion to provide further context.

**Lines 545-551**: Although *Cryptococcus* has lower frequencies of meiotic recombination compared to *S.* *cerevisiae* – approximately 1.27 crossovers per chromosome per progeny derived from intraspecific bisexual crosses [70], and this frequency is estimated to decrease by six- to seven-fold in *C. neoformans* x *C. deneoformans* hybrid progeny [46] – we expected to see significantly higher rates of recombination in hybrid progeny from *msh2*Δ crosses compared to wild-type crosses based on previous studies in both prokaryotes and eukaryotes [8,10,11].

It’s particularly interesting that the few recombination events identified are in the wild-type hybrid crosses instead of the msh2 knockouts. What might this suggest?

**Response**: We apologize for incorrectly stating in the previous submission that the few recombination events were in wild-type strains. We have changed the statements regarding this error to reflect the fact that the number of recombination events detected in the hybrid progeny were similar across the different types of hybrid crosses (wild-type, unilateral *msh2*Δ, and bilateral *msh2*Δ).

Other comments and questions
What was the intention behind creating a series of msh2 knockouts and why do the independent msh2 knockouts have variable mutation rate and other phenotypes? Is this likely because of mutations at other loci due to hypermutator phenotype?

**Response:** We generated multiple independent genetic deletion strains to confirm that the observed phenotypes were attributable to loss of Msh2 and not due to other spontaneous changes in the genomes. There is a small amount of variability in phenotypes between the independent genetic deletion mutants, and this is likely due to either experimental variability or the spontaneous mutations that are occurring throughout the genome at a relatively higher rate due to the mutator phenotype of *msh2*Δ mutants, as the reviewer suggested. In the one instance where there was a large difference in phenotypes in the mutants (inability of JEC20 *msh*2Δ-1 to produce 5-FOA resistant colonies), we were able to utilize whole-genome sequencing to identify the causal mutation of this difference. Despite the small variation between the independent genetic deletion mutants, all mutants were similar in other assessed phenotypes, with the exception of the phenotypes mediated by the single base-pair deletion in *FUR1* in JEC20 *msh*2Δ-1 (5FC and 5FU resistance).

Can you describe what you mean by self-fertility? To a naïve Cryptococcus person, I don’t know what the typical reproductive cycle is. Can a (non-hybrid) diploid not self-fertilize normally? Can they persist asexually?

**Response**: *C. neoformans* and *C. deneoformans* are both capable of asexual and sexual growth, both in the laboratory and in nature, and they exist typically as asexually growing haploid cells. Both species have bipolar mating-type systems in which a single mating-type (*MAT*) locus encodes either the *MAT***a** or *MAT*α mating-type allele. *C. neoformans* has only been observed to undergo bisexual reproduction, between cells of opposite mating types, while *C. deneoformans* is capable of both bisexual and unisexual mating, which occurs either between two cells of the same mating type or via endoreplication. Due to the large prevalence of *MAT*α strains isolated from clinical and environmental settings, *C. neoformans* and *C. deneoformans* are thought to largely reproduce through unisexual reproduction or asexually as haploids, with infrequent instances of bisexual reproduction in nature. We appreciate the reviewer asking this question and have added additional information to the introduction so that the study will be clearer for readers from all backgrounds.

**Lines 145-152**: Both species have bipolar mating-type systems in which a single mating-type (*MAT*) locus encodes either the *MAT***a** or *MAT*α mating-type allele. *C. neoformans* has only been observed to undergo bisexual reproduction, between cells of opposite mating types, while *C. deneoformans* is capable of both bisexual and unisexual mating, which occurs either between two cells of the same mating type or via endoreplication [36]. Due to the large prevalence of *MAT*α strains isolated from clinical and environmental settings, *C. neoformans* and *C. deneoformans* are thought to largely reproduce through unisexual reproduction or asexually as haploids, with infrequent instances of bisexual reproduction in nature.

On a related note, I wasn’t clear what the expected ploidy of the hybrid progeny was until the discussion.
Are the hybrids isolated from environmental/clinical samples typically diploid?

**Response**: We apologize that this was not clear earlier on in the manuscript, and we have added additional information to the introduction as well as the related results section so that readers will be aware of previous findings. *C. neoformans* x *C. deneoformans* strains isolated from the environment as well as those generated in the laboratory are highly aneuploid or near diploid.

**Lines 156-159**: Spores produced by genetic crosses between *C. neoformans* and *C. deneoformans* isolates are known to have poor germination frequencies (~5%) relative to intraspecific crosses (~80% germination), and are typically highly aneuploid or heterozygous diploids [41].

**Lines 300-303**: The hybrid progeny from both wild-type *C. neoformans* x *C. deneoformans* crosses, as well as those from unilateral and bilateral *msh2*Δ crosses, exhibited typical *Cryptococcus* hybrid genotypes: high levels of aneuploidy and inheritance of the *MAT***a** and *MAT*α mating-type alleles from both parents [41,45].

What is wild type germination frequency between strains? (non hybrid cross) What about in msh2 knockouts in non-hybrids?

**Response**: The germination frequencies of intraspecific crosses is typically quite high (~80%). We have conducted additional intraspecific crosses between *C. neoformans* and *C. deneoformans* strains as well as their corresponding *msh2*Δ mutants to address the reviewer’s questions. These results are included in Figure S3 and Table S2 and align with previously published results of relatively high germination frequencies. Interestingly loss of Msh2 significantly reduced germination frequencies of progeny derived from the bilateral *msh2*Δ intraspecific crosses in both species. This is likely due to the mutator phenotype of *msh2*Δ mutants, as has been published in other studies (Hunter et al. 1996. *EMBO J*.; Alani et al. 1994. *Genetics*.).

Figure 2A and text: Do bald basidia = sterility? Hard to tell because the PDF version of the figures is pretty blurry, but I can’t distinguish differences between panels. Could you point out the relevant mating structures (or lack there of) that I should be looking for? Is the relevant point that even though there are more bald basidia that there is higher germination?

**Response**: The reviewer is correct that while *msh2*Δ bilateral crosses produced fewer spore-bearing basidia, the spores from those crosses had higher germination frequencies. In *Cryptococcus*, we consider strains that are unable to produce any basidiospores to be sterile. Therefore, basidia with no emerging spores or spore chains, also known as “bald” basidia, would be considered sterile. We would not consider the bilateral *msh2*Δ that produced significantly greater numbers of bald basidia to be sterile because they were still able to produce many basidia with protruding chains of viable basidiospores.

Did you look at the germination frequency of the F1 hybrid progeny that produced sexual structures after incubation on YPD for 3 weeks?

**Response**: We were unable to dissect spores from the hybrid progeny that were capable of producing what appeared to be basidia and basidiospores on the YPD medium because they produced such a small number of basidiospores that were too close to the mating patch and thus inaccessible with our dissection tools.

Some other literature to include:
Bozdag et al. bioRXiv [https://doi.org/10.1101/755165](https://urldefense.com/v3/__https://doi.org/10.1101/755165__;!!OToaGQ!_AI-A3iyQdFIogGaeS2D-WABMJ-tP4oZKjCvJcqQpOi24vQI7L0q2XGCF2rnhnqB$)
Rogers et al. 2018 [https://doi.org/10.1371/journal.pbio.2005066](https://urldefense.com/v3/__https://doi.org/10.1371/journal.pbio.2005066__;!!OToaGQ!_AI-A3iyQdFIogGaeS2D-WABMJ-tP4oZKjCvJcqQpOi24vQI7L0q2XGCF89Sf0DB$)
Li et al. 2019 [https://doi.org/10.1038/s41467-019-12927-7](https://urldefense.com/v3/__https://doi.org/10.1038/s41467-019-12927-7__;!!OToaGQ!_AI-A3iyQdFIogGaeS2D-WABMJ-tP4oZKjCvJcqQpOi24vQI7L0q2XGCF6CRHB3A$)

**Response**: We thank the reviewer for directing us to these highly relevant articles and have incorporated details on these three articles and cited them within the manuscript.

**Lines 581-586**: Additionally, these results suggest alternative pathways or additional MMR components may play different or more important roles in maintaining species boundaries in *Cryptococcus* than in other previously studied organisms; proteins like the DNA helicases Mph1 and Sgs1, which have been shown to block homeologous recombination and play significant roles in chromosome nondisjunction in budding yeast, would be ideal candidates for further investigation [77–79].

**Reviewer #3**: The manuscript by Priest et al. explores the role of MSH2 and mismatch repair (MMR) in the post-zygotic reproduction barrier in hybrids between two Cryptococcus species. Hybrids between C. neoformans and C. deneoformans are readily found in the environment and in higher frequency in clinical cases. Like the Saccharomyces yeasts, there appears to be little or no pre-zygotic reproductive isolation, however these hybrids are generally sterile with relatively low germination frequencies of meiotic products, a post-zygotic barrier. The manuscript tests the role of MMR in this low fertility by knocking out MSH2 which has been shown to improve meiotic spore viability in Saccharomyces hybrids by increasing crossing over and improving segregation of chromosomes. They find that knocking out MSH2 in both species parent of hybrids increases fertility significantly, however this does not appear to be via increased crossing over during meiosis. The authors conclude that other factors must be involved in repressing meiotic recombination in these Cryptococcus hybrids.

In general, the experiments are well done and the conclusions reasonably sound. I think the authors are underselling the role of MSH2 which clearly is involved in some way in the reproductive isolation barrier and some further discussion of this is merited.

**Response**: We thank the reviewer for appreciating the quality of our studies and appreciate the reviewer’s suggestion to reframe the conclusions regarding the role of Msh2. We have adjusted the overall narrative of the manuscript to reflect that the role of Msh2 in maintaining the species boundary between *C. neoformans* and *C. deneoformans* is unexpected, and although loss of *MSH2* increases viability, a corresponding increase in recombination is not observed.

The presumed role of MMR in reproductive isolation in hybrids, based on experiments in bacteria (E. coli and S. typhimurium) and Saccharomyces (mostly S. cerevisiae and S. paradoxus with a similar level of diversity as seen here in the Cryptococcus species), is to be saturated for mismatches during strand invasion at the beginning of the recombination process, resulting in abortion of the initial recombination intermediates. This has the consequence of reducing meiotic recombination and increasing meiosis I non-disjunction due to the lack of tension on the spindle. It’s clear from the Saccharomyces studies that different MMR components may have independent roles in this barrier – pms1 and msh2 mutants in CHR III homeologous XO (Chambers et al. 1996) – despite their shared role in MMR in vegetative cells. MSH2 has also been shown to have a mismatch independent role in telomere repeat recombination which may or may not be relevant here but indicates additional roles independent of sequence divergence.

**Response**: The reviewer astutely points out the role of Msh2 as suggested by previous studies, which have also demonstrated that different MMR components have different roles and levels of impact on these events. At this point in time, we have not investigated the roles of other MMR components in maintaining the species boundary between *C. neoformans* and *C. deneoformans*. Considering the different studies that focused on the role of Msh2 in meiotic recombination in yeast, loss of Msh2 consistently had the greatest effect on the frequency of recombination between homeologous sequences compared to other MMR components. Although we cannot assume that the functions Msh2 and impact of Msh2 loss will be identical in *Cryptococcus*, these studies highlighted Msh2 as an ideal candidate for initial analysis. Investigating the roles of other MMR components in blocking meiotic recombination between *C. neoformans* and *C. deneoformans* would be a great avenue for future studies.

In the revised manuscript, we have analyzed *de novo* telomere formation in progeny derived from unilateral or bilateral *msh2*Δ intraspecific crosses. We have also included a statement on literature regarding the role of Msh2 in telomere repeat recombination and our observations may reflect this known role.

**Lines 526-532**: In the haploid intraspecific progeny, only one instance of *de novo* telomere addition was observed, and in this case only a small portion of the chromosome (~17kb) was lost, thus avoiding the deleterious effects that might be associated with the larger losses observed in the hybrid progeny if they were to happen in a haploid background. Loss of Msh2 has been shown to promote telomeric recombination in yeast, and our results suggest Msh2 might be mediating a similar anti-recombination mechanism at telomeric and other repetitive loci in *Cryptococcus* [67].

As the role in Saccharomyces and bacteria is binding mismatches in strand invasion structures and perhaps aborting them, and not in XO resolution, then it might be that in Cryptococcus the lack of MSH2 is allowing meiosis to proceed far enough to generate viable products without the resolution of interactions into XOs. Perhaps these invasions are resolved as non-crossovers, even after meiosis is completed. Given that the progeny of the bilateral msh2 hybrids are near diploid hybrids themselves, meiosis appears to result in a skipping of the reductional division which might occur when all the homeologous chromosomes are linked by strand invasions. They might not be resolvable as XOs or if they are, they may be inviable (an explanation of the reduced frequency of meiotic structures in these mutant hybrids?) such that only those that do not complete recombination are viable. This is just speculation of course and not easily addressed experimentally here for this paper, but the authors should address the issue that MSH2 does have a role in the post-zygotic barrier, but it is an unknown or unexpected role. I think this is more exciting than the conclusion that there are other repressors of meiotic recombination.

**Response**: We greatly appreciate the reviewer’s thoughtful and insightful comments on the known consequences of Msh2 loss in meiotic recombination as well as how these previous findings relate to the observations made about the hybrid *Cryptococcus* progeny in our study. We have incorporated the reviewer’s suggestions as to what might be occurring in the *msh2*Δ hybrid progeny into the discussion to provide potential explanations for the observed results. We have added additional points in the discussion to present our results in a more exciting context, demonstrating that our results were unexpected and suggestive of additional components that might play unique or stronger roles in repressing meiotic recombination in *Cryptococcus*. We have also included citations to other studies that highlight the impact of Sgs1 and Mph1 on recombination between homeologous sequences.

**Lines 502-512**: On the other hand, it is possible that without Msh2 during the hybrid meiosis, initiation of recombination between homeologous sequences will not be prevented effectively, and meiosis will therefore proceed to a degree while homeologous chromosomes are linked by strand invasions, increasing the number of viable progeny. This could also lead to increased chromosomal nondisjunction in meiosis I or potentially a skipping of the reductional meiotic division if homeologous chromosomes remain linked by strand invasions, which could explain the near diploid genomes of hybrid progeny from the unilateral and bilateral *msh2*Δ crosses. The strand invasions between homeologous chromosomes might be resolved as non-crossovers or crossovers, although the crossovers may be inviable, which could also explain the increased number of basidia lacking spore chains in the bilateral *msh2*Δ hybrid matings.

More specific comments:

The near haploid and near diploid nature of all the progeny assessed makes sense from the assessment of the tolerance of aneuploids in S. cerevisiae (Parry and Cox 1970 Genet Res 16) where viable segregants from a triploid cross were all close to euploidy. The data presented here looks similar with the wt cross exhibiting both, the heterozygous msh2 crosses exhibiting various levels of heterozygosity in the near diploid progeny with lower levels consistent with aneuploidy (2n-1). The progeny of the msh2 homozygous cross appear to be near diploid with whole complements of each parental species which is different than random segregation of chromosomes and look more like Meiosis I non-disjunction. One question – in the 3 near haploid progeny of the wt cross are the chromosomes present a mixture of each parental species?

**Response:** We thank the reviewer for pointing out this previous study and discussing how our results are in congruence. Yes, the three progeny that are nearly haploid and derived from the wild-type hybrid cross (YX1, YX3, and YX6) inherited a mixture of chromosomes from both the *C. neoformans* and the *C. deneoformans* parent (See Figure 4A and S9A). We have also incorporated the findings from this study into and their relation to the present study in the discussion.

**Lines 479-485**: Three hybrid progeny isolated from wild-type *C. neoformans* x *C. deneoformans* crosses were close to haploid and all other hybrid progeny derived were diploid or nearly diploid. Additionally, all hybrid progeny were euploid or nearly euploid, with three or fewer aneuploid chromosomes, with only one exception. These findings are similar to those from Parry and Cox, who found that *S. cerevisiae* progeny dissected from a triploid were close to euploid, which suggested that only a limited number of aneuploid chromosomes are tolerated [62].

The lack of detections of any possible increased recombination could be due to various factors. Given that the homozygous msh2 progeny were all near diploid and heterozygous across the genome, the authors did the right thing to look for cryptic XOs (in just the sequence) by looking for reads that crossed breakpoints. In general, when XOs are resolved they will be in the longer tracts of homology. Depending on the distribution of the SNPs in the two species and the range of homologous tract lengths (are there any of 200 bp long for example?), it is possible that most XOs will be in tracts longer than the reads could cover and therefore such recombination events would be missed. Alternatively, it is possible that the recombination events lead to death as is the case in S. cerevisiae where one partner of two recombining chromosomes is unresolved (Chambers 1996) resulting in a dead spore while the other partner survives. This isn’t consistent with the results presented here but all viable progeny here appear to be MI non-disjunctions which may hide such events by resolving them after meiosis.

**Response**: The reviewer correctly notes that if there are regions within the parental genomes that share complete homology for >300 bp (minimum insert size), we would not be able to detect non-crossover recombination events based on the library preparations and sequencing read lengths that were generated for the whole-genome sequencing analysis. In the revised manuscript, we have conducted additional analysis to characterize the polymorphism density across the two parental genomes. Based on our analysis, there are no tracts that are ≥300 bp with complete homology between the two parental genomes, including in an ~40kb region of the genome that shares the highest homology between the two parents, which is known as the identity island and shares 98.5% overall sequence similarity (Kavanaugh et al. *Mol Biol Evol*. 2004) (See Figure 6). A brief statement has also been included in the revised manuscript to clarify this point.

**Lines 406-409**: Additionally, no tracts ≥ 300 nucleotides in length of complete homology between both parental genomes were identified, including within the identity island, suggesting that recombination events were not being missed due to limitations of the sequencing methods used.

Line 430 – If there is little or no recombination then QTL mapping will be difficult.

**Response:** This is true and we have subsequently removed this statement from the manuscript.

Line 491 – it’s 15% divergent not 85% divergent

**Response**: We apologize for this error and have corrected the text to reflect what the reviewer has correctly pointed out.

Line 514 – loss of MSH2 clearly did reduce the boundary between these species as there was an increase in viability of meiotic products – many of these displayed transgressive sexual behaviour as well, meaning that it may be heritable. Question – were basidiospores dissected from these progeny? How viable were they?

**Response**: We were unable to dissect spores from the hybrid progeny that were capable of producing what appeared to be basidia and basidiospores on the YPD medium because they produced such a small number of basidiospores that were too close to the mating patch and thus unfortunately inaccessible with our dissection tools.
